# RPL5 deficiency-induced ribosomal stress targets a select subset of proteins and inhibits the PI3K-Akt-mTOR signaling pathway to eradicate leukemia stem cells

**DOI:** 10.1038/s41419-025-08379-1

**Published:** 2025-12-18

**Authors:** Xiaofei He, Daijiao Ye, Jiayao He, Gangfeng Ding, Zihan Lin, Yaqian Qin, Bin Zhou, Liuzhi Shi, Xuanyu Yu, Chen Meng, Min Li, Chongyun Xing, Shenmeng Gao

**Affiliations:** 1 Medical Research Center, The First Affiliated Hospital of Wenzhou Medical University, Wenzhou, Zhejiang China; 2 The Key Laboratory of Pediatric Hematology and Oncology Diseases of Wenzhou, The Second Affiliated Hospital and Yuying Children’s Hospital of Wenzhou Medical University, Wenzhou, Zhejiang China; 3 Department of Clinical Laboratory, The First Affiliated Hospital of Wenzhou Medical University, Wenzhou, Zhejiang China; 4 Department of Hematology, The First Affiliated Hospital of Wenzhou Medical University, Wenzhou, Zhejiang China

**Keywords:** Acute myeloid leukaemia, Cancer stem cells

## Abstract

Ribosomal protein L5 (RPL5) is considered a haplo-insufficient tumor suppressor by upregulating p53 expression or promoting the inactivation of c-Myc in solid tumors upon tumor initiation. However, its detailed effect and mechanism in tumor maintenance were more complicated. Particularly, the specific role of RPL5 in acute myeloid leukemia (AML) remains unclear. In this study, we found that RPL5 expression was increased in primary AML blasts compared with normal controls, regardless of *TP53* mutation. RPL5 knockdown reduced the survival and colony-forming ability of AML cells in vitro, as well as inhibited the engraftment of leukemia stem cells (LSCs) in vivo. It indicated that RPL5 was required for the survival of AML cells, especially for maintaining the stemness of LSCs. The data analysis of RNA-seq and proteomics revealed that RPL5 deficiency eradicated LSC by inducing a cellular stress response, i.e., ribosomal stress, rather than a specific function relating to RPL5. The inhibited PI3K-Akt-mTOR signaling pathway played a central role in the ribosomal stress induced by RPL5 deficiency. To investigate the selectivity of RPL5 depletion in eradicating LSCs, we found that RPL5 expression was highest in LSCs compared to AML cells and healthy controls. Moreover, ribosomal stress specifically affected transcripts with longer exon lengths and proteins with a lower isoleucine (Ile) to valine (Val) ratio. Ile and Val are glycogenic branched-chain amino acids (BCAAs) that regulate fundamental cell processes by affecting mTOR activation through BCAA metabolism. In conclusion, RPL5 depletion-induced ribosomal stress disrupted stemness maintenance by affecting BCAA metabolism in AML, specifically inhibiting the PI3K-Akt-mTOR signaling pathway, which resulted in LSC eradication.

## Facts

- Interference with ribosome biosynthesis is a potential means of AML treatment.
- RPL5 is specifically required for the stemness maintenance of LSCs.
- Ribosomal stress affects a select subset of proteins and inhibits PI3K-Akt-mTOR signaling pathway.

## Introduction

Acute myeloid leukemia (AML) is a hematological malignancy caused by abnormal differentiation of myeloid hematopoietic stem progenitor cells [1, 2]. Despite advances in diagnostic methods, therapeutic agents, and stem cell transplantation have improved the treatment, the 5-year survival rate of AML is only 30.5% [3, 4]. More than 70% of AML patients are prone to relapse, accompanied by drug resistance [5]. Leukemia stem cells (LSCs) with abnormal self-renewal ability are considered the leading cause of AML initiation, relapse, and drug resistance [6]. Therefore, how to eliminate LSCs with specificity has been in the spotlight for decades.

Ribosomal proteins determine protein biosynthesis and participate in DNA repair, cell development regulation, and other extra-ribosomal functions [7, 8]. Interference with ribosome biosynthesis is a potential means of tumor treatment by disrupting cell homeostasis [9]. Various chemotherapeutic agents, i.e., oxaliplatin, cisplatin, actinomycin D, and 5-FU, have been identified as having antitumor effects due to disrupting ribosome biosynthesis [10, 11]. Moreover, ribosomal proteins RPS2 and RPL19 were developed as new therapeutic targets for prostate cancer, while knockout of RPS9 induced p53-dependent cell cycle arrest and differentiation in glioma cells [12, 13]. RPL39 deletion affected the self-renewal of breast cancer stem cells through the nitric oxide synthase pathway [14].

Ribosomal protein L5 (RPL5) is a 5S ribosome protein complex component and is directly involved in formatting the 60S ribosome large subunit [15]. Its somatic mutations were observed in 20–40% of patients with advanced multiple myeloma and increased the risk of T-cell acute lymphoblastic leukemia [16]. The high expression of RPL5 was associated with poor prognosis in patients with relapsed multiple myeloma and reduced cell sensitivity to bortezomib [17–19]. In addition, RPL5 germline mutations occurred in Diamond-Blackfan anemia (DBA), and the incidence of AML in DBA patients was 28–36 times higher than that in the general population [14, 20]. These implied complicated roles of RPL5 in hematologic malignancies; however, the detailed function of RPL5 in AML is still unknown.

Herein, we adopted cell and murine models to investigate whether RPL5 expression affected AML progression and found that RPL5 was highly expressed in AML cells compared with normal controls (NC). *RPL5* knockdown inhibited AML cell survival in vitro and in vivo, especially destroying the stemness maintenance of LSCs, which could be exploited as a therapeutic target for AML. Moreover, RNA-seq, proteomics, and molecular studies were performed to uncover the mechanism by which RPL5 deficiency-induced ribosomal stress affected branched-chain amino acid (BCAA) metabolism, specifically inhibiting PI3K-AKT-mTOR signaling pathway, resulting in LSCs eradication.

## Materials and methods

### Cell culture

AML cell lines (ATCC, USA) were cultured in a 37 °C humidified incubator with 5% CO_2_ in RPMI-1640 with 10% or 20% fetal bovine serum (FBS12B020, ExCell Bio) and 1% penicillin/streptomycin (C100C5, NCM Biotech). Primary AML blasts were isolated by Ficoll density gradient centrifugation (GE Healthcare, USA) and human normal CD34^+^ cells were isolated with biotin mouse anti-human CD34 antibody (#564669, BD PharMingen). Primary AML blasts were cultured in SFEM Medium (Stemcell Technologies, Canada) supplemented with recombinant human SCF, IL-3, and IL-6 (PeproTech, USA) and normal CD34^+^ cells were cultured in SFEM supplemented with human TPO, Flt3 ligand, and SCF (PeproTech, USA) at 100 ng/mL each. Both AML patients and healthy volunteers have provided informed consent according to the Declaration of Helsinki. The clinical characteristics of AML patients were summarized in Table S1. All procedures followed the Declaration of Helsinki and the Ethics Committee of the First Affiliated Hospital of Wenzhou Medical University (WMU), and no mycoplasma contamination was observed upon routine identification.

### Lentivirus and retrovirus production and transfection

Retroviral vector MSCV-GFP-AF9, together with Gap pol and VSVG, or lentiviral vector pLKO-NC/RPL5 with MD2G and psPAX2, were respectively mixed with linear polyethyleneimine (PEI, Sigma-Aldrich) and co-transfected into plated HEK293T cells for 48 and 72 h. The collected supernatant was filtered (FPE-404-030, JET BIOFIL) and then mixed with polybrene (HY-112735, MCE) for AML cell transfection. Green fluorescent protein (GFP) or puromycin (ant-pr-1, InvivoGen) was used to select positive clones.

### Xenograft mouse model

MOLM-13 cell line (1 × 10^6^ cells) transfected with lentivirus expressing shNC or shRPL5 (2 days post-transduction) was implanted into lethally irradiated NOD-scid IL2Rγnull (NSG) mice by tail vein injection. NSG mice exhibiting no visible abnormalities in appearance or behavior were randomly assigned to control and experimental groups. We strictly followed statistical methods for sample size determination, adhered to the 3R principles in animal experiments, and provided detailed sample size information in figure legends. A small amount of blood was collected from the posterior orbital venous plexus every two weeks to detect the proportion of human CD45^+^ (hCD45^+^) cells in peripheral white blood cells by flow cytometry. Engraftment was generally considered successful when the percentage of hCD45^+^ cells exceeded 1%. Recipient mice were monitored for survival. All animal experiments were conducted in accordance with the ARRIVE guidelines 2.0 and approved by national and international policies and institutional guidelines of the ethics committee at WMU (blinded for review). Every effort was made to minimize animal suffering.

### AML_*MLL-AF9*_ murine model

Mouse hematopoietic progenitor cells (EasySep^TM^ Mouse Hematopoietic Progenitor Cell Isolation Kit, #19856A, Stem cell Technologies) were isolated from the BM of 8-week-old C57BL/6 mice and transduced with MSCV-GFP-IRES-MLL-AF9 retrovirus [21]. GFP^+^ cells were sorted by fluorescence-activated cell sorting (FACS) and were intravenously injected into lethally irradiated C57BL/6 mice (Beijing Vital River Laboratory, China). When mice developed AML, GFP^+^ BM cells were isolated and transfected with lentivirus for *Rpl5* knockdown. Puromycin-selected cells were mixed with supportive cells and then intravenously injected into lethally irradiated C57BL/6 mice for primary bone marrow transplantation (BMT). The BM cells of primary mice mixed with supportive cells were injected into lethally irradiated C57BL/6 mice for secondary BMT. The BM cells of secondary mice were isolated and injected into lethally irradiated C57BL/6 mice for tertiary BMT. C57BL/6 mice exhibiting no visible abnormalities in appearance or behavior were randomly assigned to control and experimental groups. All animal procedures and care were performed according to national and international policies and institutional guidelines of the Ethics Committee of the First Affiliated Hospital of WMU.

### Limiting dilution assay

Series indicated doses of donor cells were sorted from shNC or shRpl5 AML_*MLL-AF9*_ secondary mice and mixed with supportive cells, which were transplanted into lethally irradiated recipients. Count the number of recipient mice only when they developed full-blown leukemia and died within 4 months after transplantation [22].

### Flow cytometry analysis

PB cells were collected and lysed with RBC lysis buffer first. BM cells were isolated by crushing bones and stained with biotin mouse lineage panel for 30 min (#6209979, BD PharMingen). Then leukemia initiation cells (LIC) [23] were stained with c-Kit and Mac-1, while L-GMP cells were stained with specific antibodies including Streptavidin, Sca-1, CD34, c-Kit, CD16/CD32, Mac-1 and CD135 (BD PharMingen, USA). Thymidine analog 5-ethynyl-2’-deoxyuridine (EdU) [24] staining was performed following a standard protocol (C0081S, Beyotime Biotechnology). To examine cell death and apoptosis, AML cells expressing shNC or shRPL5 were stained with Annexin V-APC and 7-AAD kit according to the manufacturer’s instructions (AP105-100-kit, Multi Sciences). To examine the cell cycle, Hoechst33342 and Pyronin Y were used for LIC staining. All experiment was performed by CytoFLEX LX (Beckman-Coulter, USA), and cell sorting was performed by FACS Aria II (Becton Dickinson, USA). Data were analyzed with FlowJo software v10.0.

### Colony-forming unit (CFU) assay

AML cells (2 × 10^5^/mL) were transduced with lentiviruses encoding shNC or shRPL5 for two days. After 1 μg/mL puromycin selection, 2000 AML cells expressing shNC or shRPL5 were plated in 1 mL of methylcellulose medium (MethoCult^TM^ H4434 Classic, StemCell Technologies) and placed in 35 mm culture dishes with triplicate wells. Colonies were counted after 10 days using a Leica DMi1 microscope (Leica, Germany). Primary mouse cells, which performed lineage depletion (EasySep^TM^ Mouse Hematopoietic Progenitor Cell Isolation Kit, #19856A, Stem cell Technologies), were plated in 1 mL of methylcellulose medium (MethoCult^TM^ GF M3434, StemCell Technologies) and placed in 35 mm culture dishes with triplicate wells. The number of colonies was counted after 14 days at 37 °C.

### Measurement of cell growth

Leukemic cells expressing shNC or shRPL5 were seeded in 96-well plates. After plating for 48 h, CCK-8 solution (CK04, Dojindo) was added and incubated for 2 h. The absorbance was measured at 450 nm by an MRX II microplate reader (Dynex, USA).

### Immunofluorescence assay

AML cells were collected onto slides by cytospin (ELITech, USA) and fixed in 4% paraformaldehyde, followed by permeabilization with 0.2% Triton X-100 in PBS. After leukemic cells were blocked with 5% BSA in PBS for 2 h at room temperature, the cells were incubated with 4,6-diamidino-2-phenylindole (1 μg/mL, P0131, Beyotime Biotechnology) solution. Fluorescence signals were detected on a laser scanning confocal microscope (Olympus-FV3000, Japan).

### Transmission electron microscope assay

Cell samples were fixed by immersion in glutaraldehyde solution followed by rinsing with cold PBS Buffer (PH 7.4). Then, samples were fixed with an osmic acid solution, and an increasing concentration of acetone was used for dehydration. After that, samples were embedded and polymerized for the ultrathin section, which was examined by TEM (Hitachi H-7500, Japan).

### Western blotting

Leukemic cells were harvested and lysed completely by RIPA lysis buffer following standard procedure (P0013B, Beyotime Biotechnology), and the concentration was measured by a BCA assay (P0009, Beyotime Biotechnology). After that, 20 μg/well proteins were fractionated by electrophoresis through polyacrylamide gels and transferred to a PVDF membrane (Millipore, USA). After blocking with TBS containing 5% skim milk powder, blots were incubated with primary antibodies overnight at 4 °C and then incubated with a second HRP-conjugated antibody for 1 h (Table S2). Signals were measured by chemiluminescence reagents using an imaging system (Bio-Rad, USA).

### Quantitative RT-PCR

Total mRNA was extracted by Trizol reagent according to the manufacturer’s instructions (RNAiso Plus-9109, Takara). The concentration and quality were determined by measuring the absorbance at 260/280 nm with a spectrophotometer (DS-11 spectrophotometer, DeNovix). cDNA was synthesized through Q5 real-time PCR system (Applied Biosystems, USA) and used as a template for qRT-PCR through SYBR Green dye (RR820A, Takara) on Mastercycler real-time PCR system (LightCycler, Roche). Relative expression was calculated using the 2^–ΔΔCT^ method. All the primer sequences were shown in Table S3.

### Statistical analysis

Unless otherwise noted in Results and figure legends, statistical significance was evaluated using an unpaired two-tailed Student’s *t* test when comparing the difference between two samples, and one-way ANOVA was employed when comparing the samples among groups with more than two samples with GraphPad Prism (v10, GraphPad). The Mantel-Cox test was used to analyze the Kaplan-Meier survival curve. Results were shown as the Mean ± SD. The analysis of sequencing result, R script used in the manuscript, the raw data of qRT-PCR, as well as the genotyped matrix of damaging mutation relating to the RPL5 gene in cell lines were deposited in GitHub public repository (https://github.com/Fang-He1111/RPL5).

## Result

### RPL5 is highly expressed in AML cells with different karyotypes

**Fig. 1:**
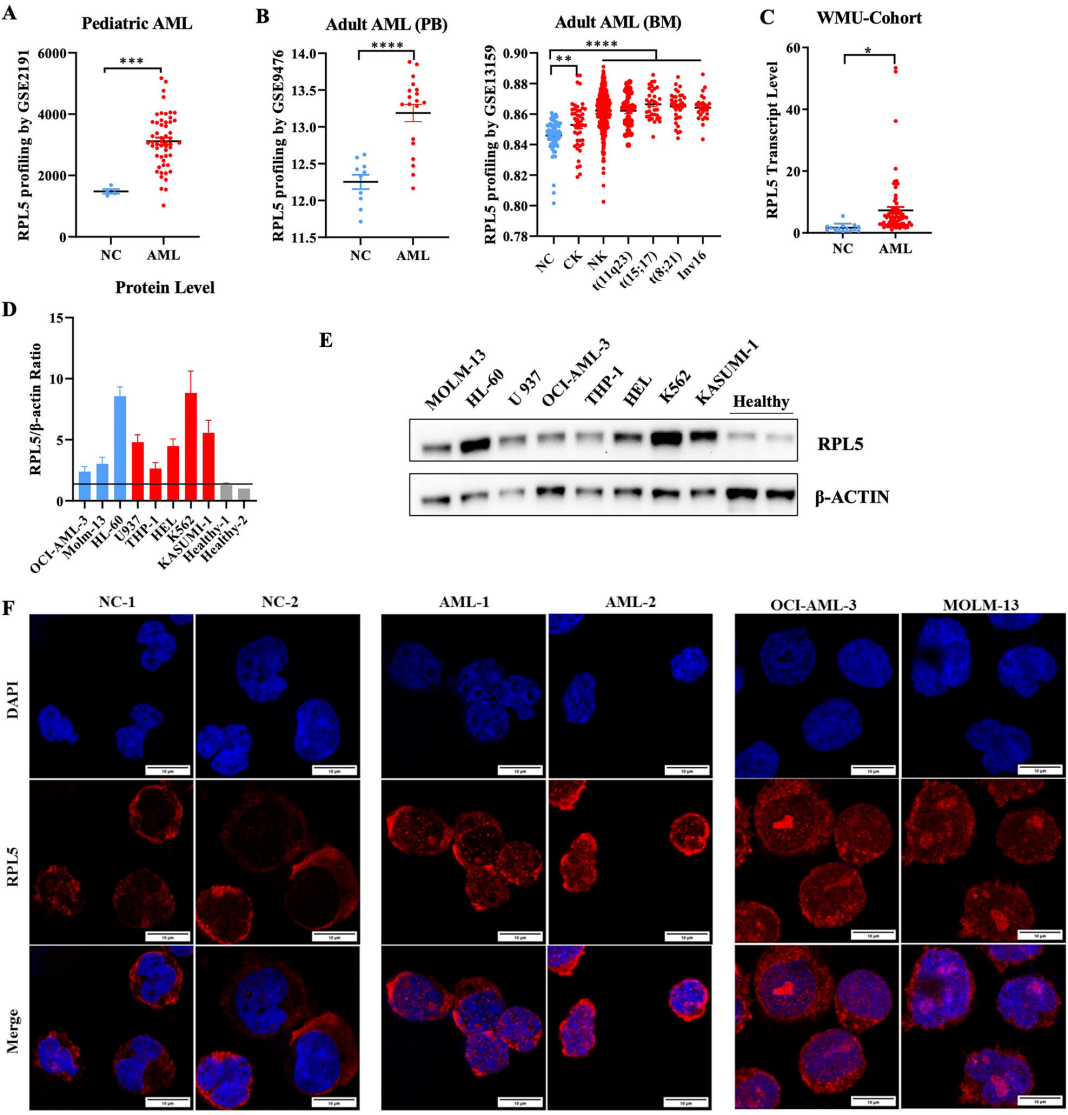
RPL5 is highly expressed in AML. **A** Levels of RPL5 mRNA in BM MNC from pediatric AML (*n* = 54) and age-matched normal controls (*n* = 4). **B** Levels of RPL5 mRNA in PB (*n* = 19) and BM (*n* = 501) based on different karyotypes from adult AML and age-matched normal controls (*n* = 10 and *n* = 73, respectively). **C** Levels of RPL5 mRNA in BM MNC of AML patients (*n* = 74) and age-matched normal controls (*n* = 14) of WMU-Cohort. Summarized bar-graph (**D**) and representative WB picture (**E**) of RPL5 protein levels in AML cell lines compared with BM MNC from healthy controls. **F** Representative confocal pictures of RPL5 expression in BM MNC from normal controls, patients, and cell lines. (*** *P* < 0.001, **** *P* < 0.0001).

To explore the connection of RPL5 in human leukemia, published genomic studies on AML were analyzed using cBioPortal (https://www.cbioportal.org/) first. Although RPL5 mutations were frequently observed in multiple myeloma and DBA, only three amplifications and one deep deletion pertain to *RPL5* mutations among 6205 AML samples from 5340 patients in 8 studies (Updated 2025-09). Therefore, the mRNA expression of RPL5 in AML patients was analyzed through public databases GSE2191, GSE9476, and GSE13159, respectively [25–27]. Compared with age-matched NC, RPL5 mRNA levels were enhanced in mononuclear cells (MNC) from bone marrow (BM) of pediatric AML patients (Fig. 1A), as well as MNC from peripheral blood (PB) and BM of adult AML patients (Fig. 1B). Although the RPL5 expression of AML patients with complex karyotypes (CK) was lower than other AML subgroups, it was still higher than the healthy controls, indicating that RPL5 was highly expressed in AML with different karyotypes compared with healthy controls (Fig. 1B).

Consistently, increased transcript levels of RPL5 were also observed in the WMU-cohort of AML patients with different karyotypes compared with healthy controls (Fig. 1C and Table S1). Additionally, we determined the protein levels of RPL5 in AML and found that AML cell lines with different karyotypes and mutations exhibited enhanced RPL5 protein levels compared to healthy controls (Fig. 1D, E). The enhanced RPL5 expression was particularly enriched in the cell nucleus of AML patient samples and AML cell lines (Fig. 1F). Considering *TP53* mutation may affect RPL5 expression, we chose OCI-AML-3 and MOLM-13, harboring intact *TP53*, as well as HL-60, harboring homozygous *TP53* deletion, for later functional investigation (Fig. 1D and Table S4). Notably, no damaging mutations related to the RPL5 gene were found in the AML cell lines mentioned above, as per a search of the Depmap Portal (Table S5, https://sites.broadinstitute.org/ccle/tools).

### RPL5 is required for maintaining AML cell survival in vitro and in vivo

**Fig. 2:**
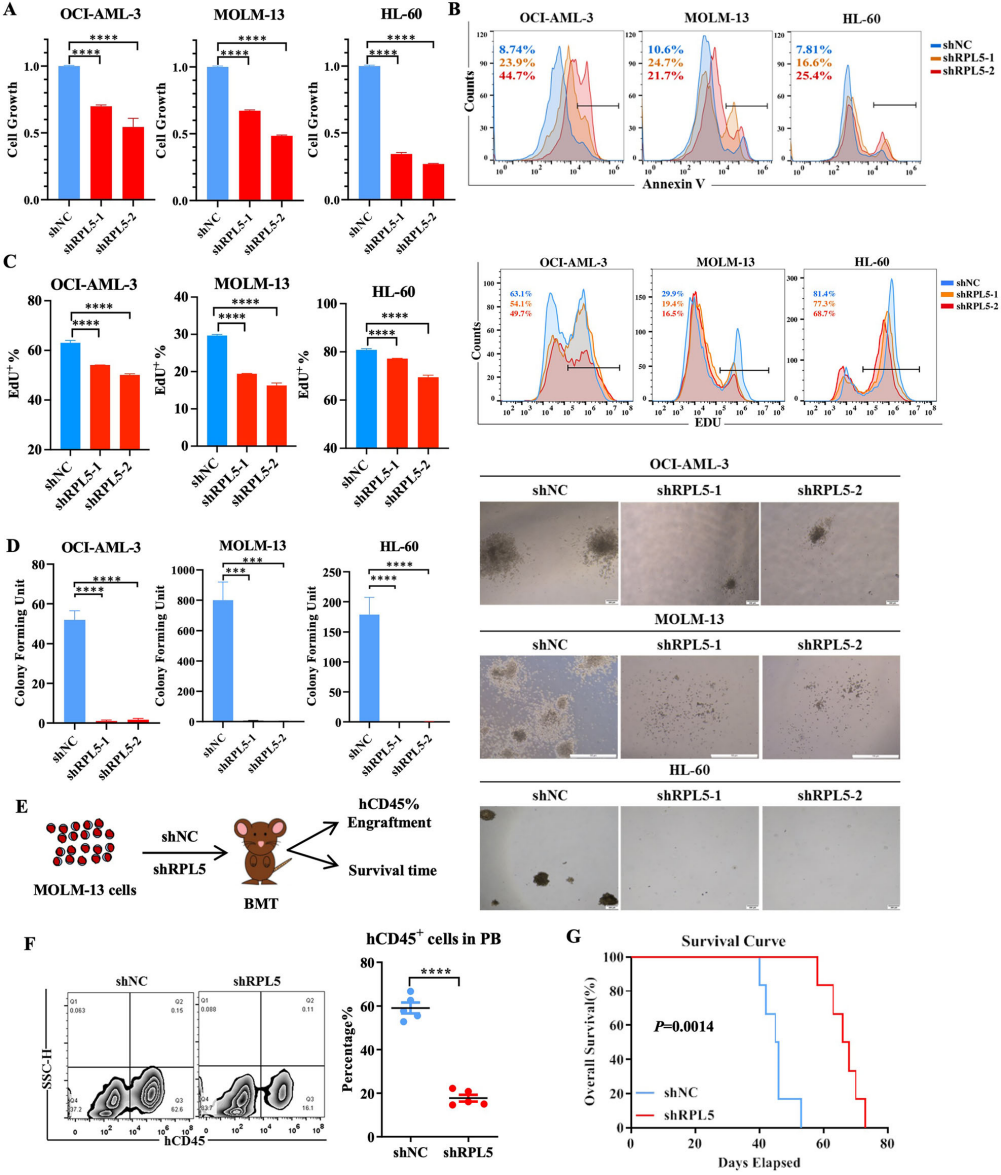
RPL5 is required for AML cell survival. **A** Cell growth of AML cells expressing shNC or shRPL5 was measured using the CCK8 assay (*n* = 3/group). **B** Representative figure of Annexin V^+^ cells in AML cells expressing shNC or shRPL5. **C** Representative figure and flow cytometric analysis of EdU^+^ cells in AML cells expressing shNC or shRPL5. **D** Analysis and representative figures of colonies produced by AML cells expressing shNC or shRPL5 (*n* = 3/group). **E** Schematic picture of the cell-derived xenografted model. **F** Representative figure and flow cytometric analysis of hCD45^+^ engraftment in PB of recipient mice xenografted with MOLM-13 cells expressing shNC or shRPL5 (*n* = 5/group). **G** Kaplan-Meier curve of recipient mice xenografted with MOLM-13 cells expressing shNC or shRPL5 (*n* = 6/group). (***P < 0.001, ****P < 0.0001).

Since RPL5 was overexpressed in AML, RNA interference (shRNA) targeting RPL5 was expected to destroy AML cell survival. Thus, two independent shRNAs, shRPL5-1 and shRPL5-2, were designed and significantly reduced the transcript and protein levels of RPL5 compared to the control shRNA (shNC) in AML cell lines (Fig. S1A, B). As a result, the cell growth was inhibited in two shRPL5 groups compared with the shNC group in all AML cell lines, regardless of *TP53* mutation (Fig. 2A). This indicated that RPL5 regulated AML cell growth independently of *TP53* states. This finding was consistent with the public database analysis (GSE132245 and GSE131592), which showed that the depletion of *TP53* did not affect RPL5 gene expression in OCI-AML-3 and MOLM-13 cells (Fig. S1C, D). Moreover, AML cells expressing shRPL5 exhibited an increased frequency of Annexin V^+^ cells compared with those expressing shNC (Fig. 2B), indicating enhanced apoptosis in AML cells due to the depletion of RPL5. The decreased transcript and protein levels of BCL2 were in accordance with the increased cell apoptosis (Fig. S1E, F).

Besides, RPL5 knockdown inhibited cell proliferation, as represented by reduced EdU^+^ staining in the shRPL5 groups compared with the control group (Fig. 2C). More AML cells were blocked in the G1 stage due to RPL5 knockdown (Fig. S2A). To further examine the effect of RPL5 on leukemia progenitor cell function, AML cells expressing shNC or shRPL5 were plated in methylcellulose for the CFU assay. Intriguingly, AML cells expressing shRPL5 almost lost their colony-forming capacity (Fig. 2D), indicating a more critical role of RPL5 in maintaining the stemness of LSC compared with regulating cell growth and cell apoptosis. Although RPL5 deficiency inhibited the colony formation of normal CD34^+^ primary cells, it was not as severe as it was for LSCs (Fig. S2B). In addition, to investigate the efficacy of RPL5 depletion on AML cell engraftment in vivo, MOLM-13 cells expressing shNC or shRPL5 were xenografted into immunocompromised NSG mice (Fig. 2E). A large amount of hCD45^+^ leukemia cells were observed in the PB of recipient mice in the shNC group but less in the shRPL5 group (Fig. 2F). Starting from 6 weeks, the recipient mice of the shNC group succumbed to lethality with a median survival time of 45 days (Fig. 2G), and the recipient mice of the shRPL5 group exhibited extended survival time (67 days), indicating a supportive role of RPL5 in AML cell expansion in vivo.

### Rpl5-deficiency impedes AML progression in the murine model

**Fig. 3:**
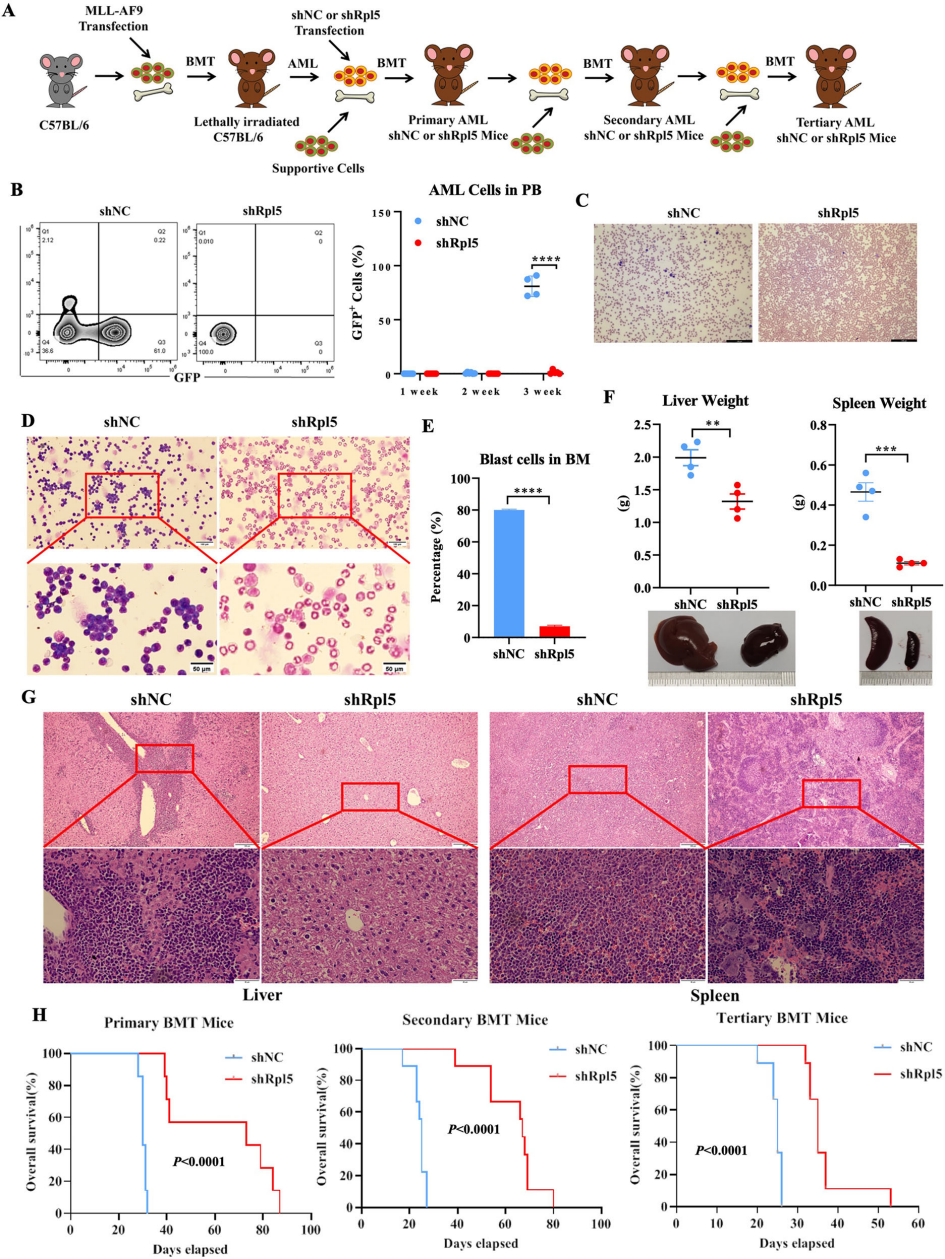
RPL5-deficiency hinders AML progression in the murine model. **A** Schematic figure of *MLL-AF9*-transformed AML murine model. **B** Flow cytometric analysis and representative picture of GFP^+^ AML cells engraftment in PB of secondary recipients expressing shNC or shRpl5 (*n* = 4). Wright-Giemsa staining of AML blast in PB (**C**) and BM (**D**) of secondary recipients expressing shNC or shRpl5. **E** Cell counting analysis of Wright-Giemsa-stained AML blast in BM of secondary recipients expressing shNC or shRpl5 (*n* = 4/group). **F** The weight and representative picture of liver and spleen from secondary recipients expressing shNC or shRpl5 (*n* = 4/group). **G** The H&E stain of the liver and spleen slides of secondary recipients expressing shNC or shRpl5. **H** Kaplan-Meier curve of recipient mice engrafted with AML_*MLL-AF9*_ cells expressing shNC or shRpl5 (*n* = 9 mice/group). (**P < 0.01, *** *P* < 0.001, **** *P* < 0.0001).

To further understand the supportive role of RPL5 in AML progression, an Rpl5-deficient *MLL-AF9*-transformed AML murine model was constructed and verified by reduced transcript and protein levels of the target gene (Figs. 3A and S3A, B). As a result, there were more engrafted GFP^+^ leukemia cells in the PB of secondary recipients expressing shNC, and leukemic cells were hardly detected in recipients expressing shRpl5 (Fig. 3B, C). Consistently, reduced AML blasts were also observed in the BM of shRpl5 recipients compared with those of shNC recipients (Fig. 3D, E). Moreover, Rpl5 deficiency inhibited the extramedullary invasion of AML cells, as evidenced by alleviated hepatosplenomegaly and improved pathological features in the shRpl5 group compared with the shNC group (Fig. 3F, G).

The overall improved AML features induced by Rpl5 depletion finally extended the median survival time of primary recipients for more than 40 days compared with the shNC group (73 days vs. 30 days, Fig. 3H), while a similar tendency was continued with secondary recipients (67 days vs. 25 days, Fig. 3H). Considering the significant inhibition of RPL5 deficiency on leukemia progenitor cells, BM cells isolated from secondary recipients were used for constructing a tertiary murine model to examine the long-term effect of Rpl5 deficiency on LSCs. With consistency, the tertiary generation still showed extended survival time (35 days vs. 25 days, Fig. 3H), emphasizing a specific role of Rpl5 in LSCs, which could be exploited as a therapeutic target.

### Rpl5 deficiency inhibits LSCs survival through a G0-phase cell cycle arrest

**Fig. 4:**
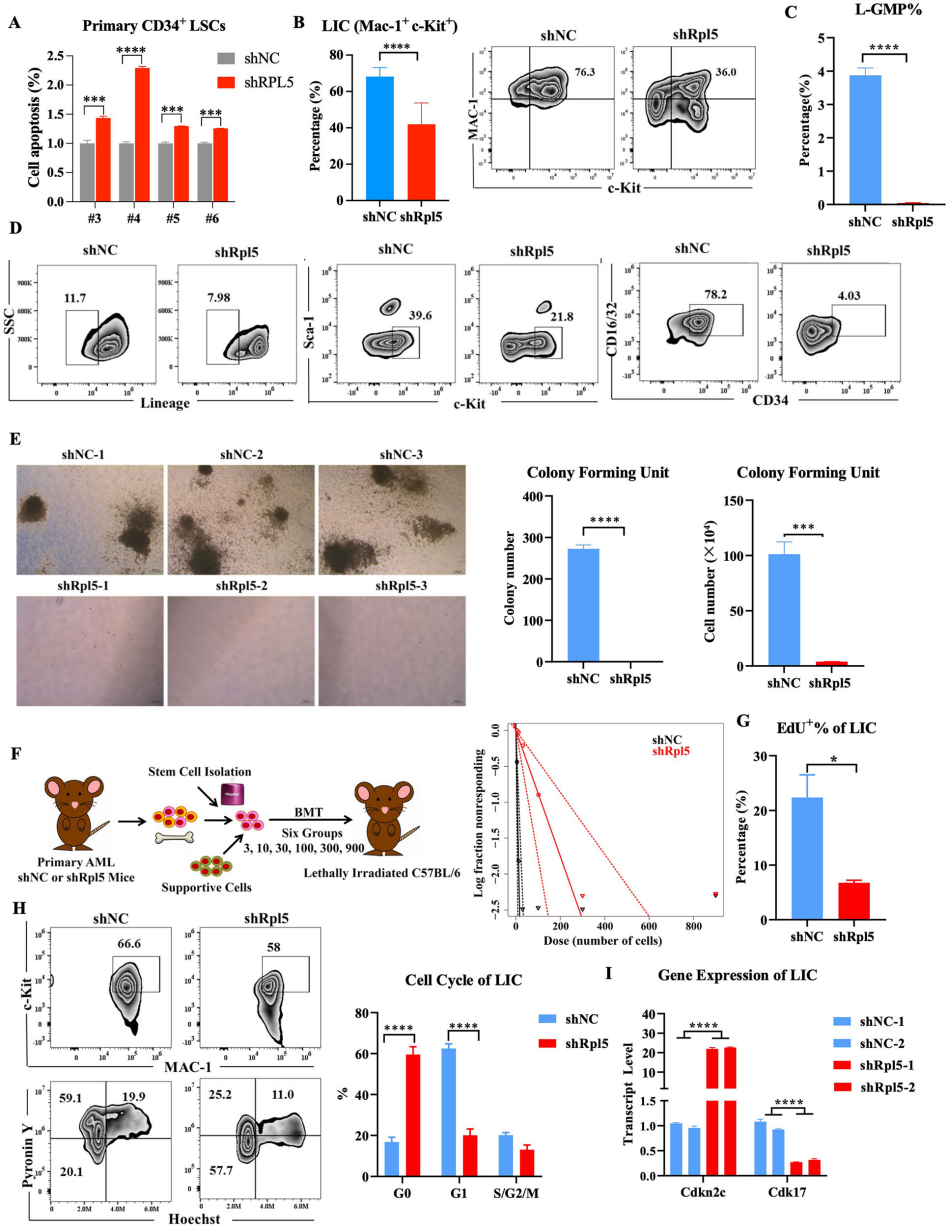
RPL5 deficiency inhibits LSC frequency and function through a G0-phase cell cycle arrest. **A** Relative cell apoptosis of primary CD34^+^ LSCs expressing shNC or shRPL5 (*n* = 3/group). **B** Representative picture and the flow cytometric analysis of LICs in BM from secondary recipients expressing shNC or shRpl5 (*n* = 4/group). The flow cytometric analysis (**C**) and representative picture (**D**) of L-GMP in BM from secondary recipients expressing shNC or shRpl5 (*n* = 4/group). **E** Representative figure, colony, and total cell number produced by LK cells from secondary recipients expressing shNC or shRpl5 (*n* = 3/group). **F** Schematic picture and ELDA analysis of recipients with diluted LK cells expressing shNC or shRpl5 (*n* = 6/group). **G** Percentage of EdU^+^ BM LICs from secondary recipients expressing shNC or shRpl5 (*n* = 4/group). **H** Representative picture and the flow cytometric analysis of the cell cycle of BM LICs from secondary recipients expressing shNC or shRpl5 (M, *n* = 4/group). **I** *Cdkn2c* and *Cdk17* transcript levels in BM GFP^+^ cells from secondary recipients expressing shNC or shRpl5 (*n* = 3/group). (*P < 0.05, **P < 0.01, ****P < 0.0001).

Since RPL5 depletion significantly reduced the colony-forming ability and extended the survival time of AML mice, this implied that RPL5 regulated the maintenance of stemness in LSCs. RPL5 deficiency induced significant cell apoptosis in primary CD34^+^ LSCs (Fig. 4A). To specify the role of RPL5 in stem cells, the percentage of LSCs in mice represented by LIC and L-GMP subpopulations was examined. Both ratios of LIC and L-GMP subpopulation were decreased in the shRpl5 group compared with the shNC group (Fig. 4B–D). At the same time, most of the reduced L-GMP could be attributed to the reduced CD34 expression (Fig. 4D). Correspondingly, the Mac-1^+^Gr-1^+^ marked differentiated AML cells were increased after the depletion of Rpl5 (Fig. S4A).

To further illustrate the functional change of LSCs, Lin-c-Kit+ (LK) cells enriched with stem and progenitor cells were sorted from the BM of secondary recipient mice for the CFU assay. LK cells of the shRpl5 group formed a few colonies with reduced total cell number compared with the shNC group (Fig. 4E), suggesting a defective function of RPL5-deficient LSCs. Meanwhile, the limited-diluted assay showed that Rpl5-deficient LSCs prolonged the time of developing full-blown AML compared to controls, when 4 months was used as the endpoint (Fig. 4F and Table S6). The original cell apoptosis was negligible in both the shNC and shRpl5 groups (Fig. S4B). In brief, Rpl5 deficiency eradicated LSCs by simultaneously impairing their frequency and function. At the same time, it did not inhibit the colony formation of normal hematopoietic stem and progenitor cells (HSPCs) of mice (Fig. S4C, D).

After that, the EdU staining was performed to uncover cellular mechanisms relating to the RPL5 deficiency-impaired LSC. It showed that Rpl5 deficiency reduced the proliferation index of LIC but not GFP^+^ AML cells compared with the shNC group (Figs. 4G and S4E), revealing an inactive DNA synthesis in Rpl5-deficient LSCs. Furthermore, the Pyronin Y and Hoechst staining identified an elevated G0 phase in the LIC of the shRpl5 group compared with the shNC group (Fig. 4H), which was double-checked with the combination of Ki-67 and Hoechst staining (Fig. S4F). Moreover, the gene expression of cyclin-dependent kinase inhibitor 2C (Cdkn2c), which interacts with Cdk4/6 and controls cell cycle G1 progression, was augmented in the shRpl5 group compared with the shNC group (Fig. 4I) [28]. Cyclin-dependent kinase 17, which belongs to the subfamily of the Ser/Thr family of protein kinases, was also reduced during Rpl5 deficiency (Fig. 4I) [29]. These data indicated that the Rpl5 deficiency inhibited LSCs through a G0-phase cell cycle arrest.

### RPL5 deficiency inhibits LSCs by inducing ribosomal stress

Regarding the molecular mechanism, previous studies have reported that RPL5 cooperates with RPL11 and binds to double minute homolog 2 (MDM2 or HDM2), which can reduce the degradation of p53 by the ubiquitination ligase E3, thereby initiating cell cycle arrest and apoptosis [30]. It also promotes RNA-induced silencing complex-mediated degradation of c-Myc mRNA and inhibits c-Myc protein activity [31]. However, RPL5 depletion in AML cells did not show a consistent effect on p53 or c-Myc protein levels, indicating that RPL5 deficiency did not depend on p53 or c-Myc-related pathways to affect the stemness maintenance of LSC (Fig. S5A). It was consistent with our previous observation that *TP53* mutation did not directly affect RPL5 expression in AML. Since RPL5 is a constitutive element of the 60S ribosomal large subunit, interfering with the biosynthesis of RPL5 may trigger ribosomal stress and inhibit tumor cell proliferation directly [15].

**Fig. 5:**
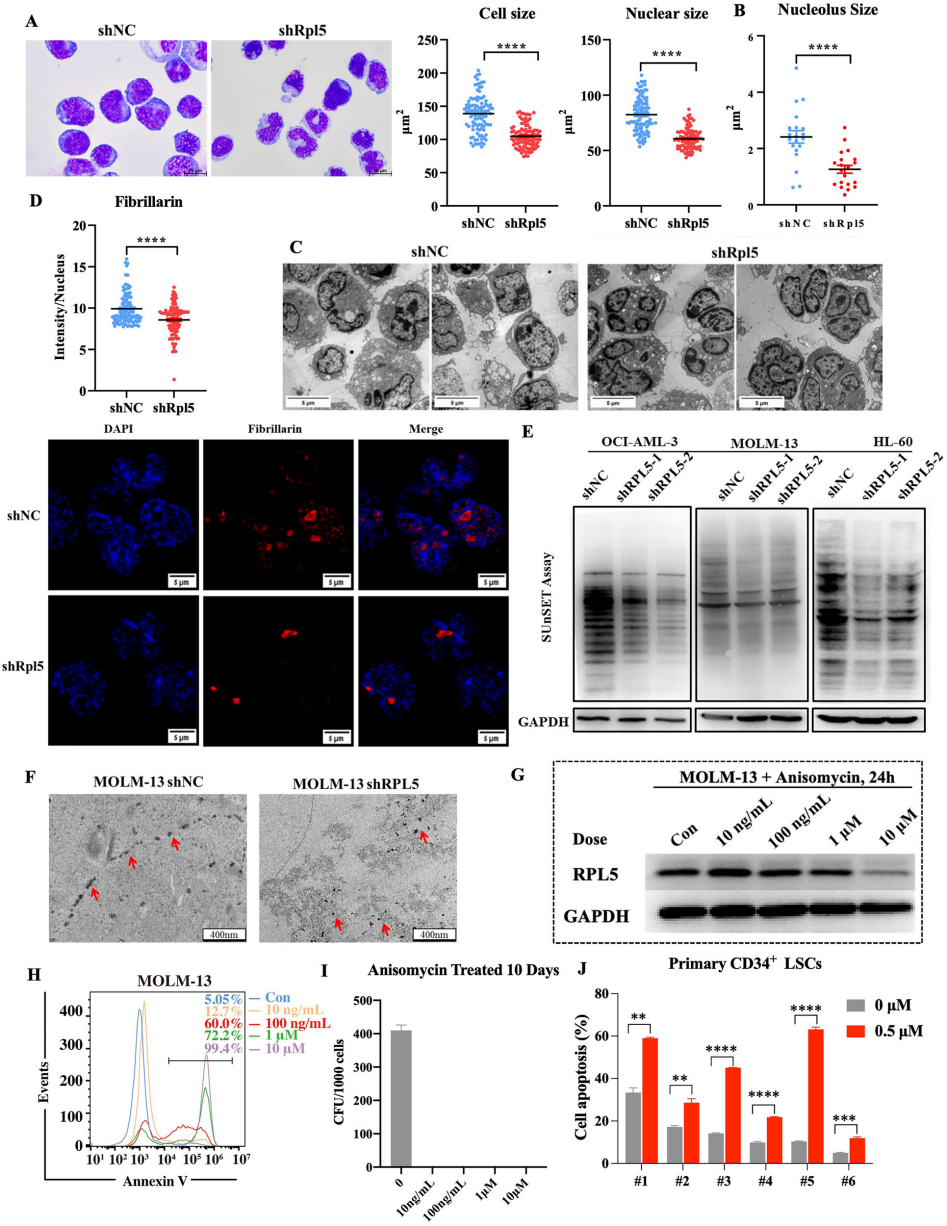
RPL5 deficiency induces ribosomal stress. **A** Wright-Giemsa staining and the quantification of cell size and nuclear size of AML blasts from secondary recipients expressing shNC or shRpl5 (*n* = 100/group). The quantification (**B**) and representative pictures (**C**) of nucleolus size of GFP^+^ BM cells from secondary recipients expressing shNC or shRpl5 (*n* = 20/group). **D** The quantification and representative pictures of fibrillarin expression in GFP^+^ BM cells of secondary recipients expressing shNC or shRpl5 (*n* = 100/group). **E** Protein synthesis rate of AML cells expressing shNC or shRPL5. **F** TEM picture of MOLM-13 cells expressing shNC or shRPL5. **G** The protein level of RPL5 in MOLM-13 cells treated with Anisomycin. **H** The cell apoptosis of MOLM-13 cells treated with Anisomycin. **I** The colonies produced by MOLM-13 cells treated with Anisomycin for 10 days (*n* = 3/group). **J** The cell apoptosis of primary CD34^+^ LSCs treated with 0.5 μM Anisomycin for 24 h (*n* = 3/group). (**P < 0.01, ***P < 0.001, ****P < 0.0001).

To confirm this conjecture, we examined the ribosomal stress in the murine model and found that the cell size and nuclear size of AML blasts were decreased in the shRpl5 group compared with the shNC group in Wright staining (Fig. 5A). The TEM pictures showed reduced nucleolus size and the nucleus morphology of GFP^+^ BM cells was also changed with a more visible boundary in the shRpl5 group compared with the shNC group (Fig. 5B, C). Consistent with previous findings that RPL5 deficiency led to LSC eradication, the original round or oval-shaped nucleus of AML blast changed into an irregular mature nucleus upon RPL5 deficiency (Fig. 5A, C). Besides, the intensity of Fibrillarin, a specific nucleolus biomarker [31], was decreased in BM cells of the shRpl5 group (Fig. 5D). Furthermore, Rpl5 deficiency-induced ribosomal stress spread to the ER. Reduced ribosome attachment and altered ER architecture were observed in TEM images (Fig. S5B). With consistency, RPL5 deficiency in AML cell lines also induced ribosomal stress, as represented by reduced protein synthesis and random ribosome distribution when compared with the control group (Fig. 5E, F).

To verify the theory that RPL5 deficiency eradicated LSC by inducing a cellular stress response, i.e., ribosomal stress, rather than affecting a specific role related to RPL5 expression, we used Anisomycin, a protein synthesis inhibitor, to induce moderate or severe ribosomal stress at different concentrations [32, 33]. Low-dose Anisomycin (10 ng/mL)-induced moderate ribosomal stress did not affect RPL5 expression, while high-dose Anisomycin (10 μM)-induced severe ribosomal stress inhibited RPL5 expression (Fig. 5G). Although 10 ng/mL Anisomycin slightly induced cell apoptosis (Fig. 5H and S5C), it significantly inhibited the colony formation of AML cells at 10 days and 15 days (Fig. 5I and S5D, E). Therefore, RPL5 deficiency eradicated LSC by inducing ribosomal stress rather than affecting a specific role relating to RPL5. Additionally, low-dose Anisomycin (0.5 μM) induced dramatic cell apoptosis in primary CD34^+^ AML stem cells, demonstrating its potential clinical application (Fig. 5J).

### PI3K-AKT-mTOR signaling pathway plays a critical role in RPL5 deficiency-induced ribosomal stress

**Fig. 6:**
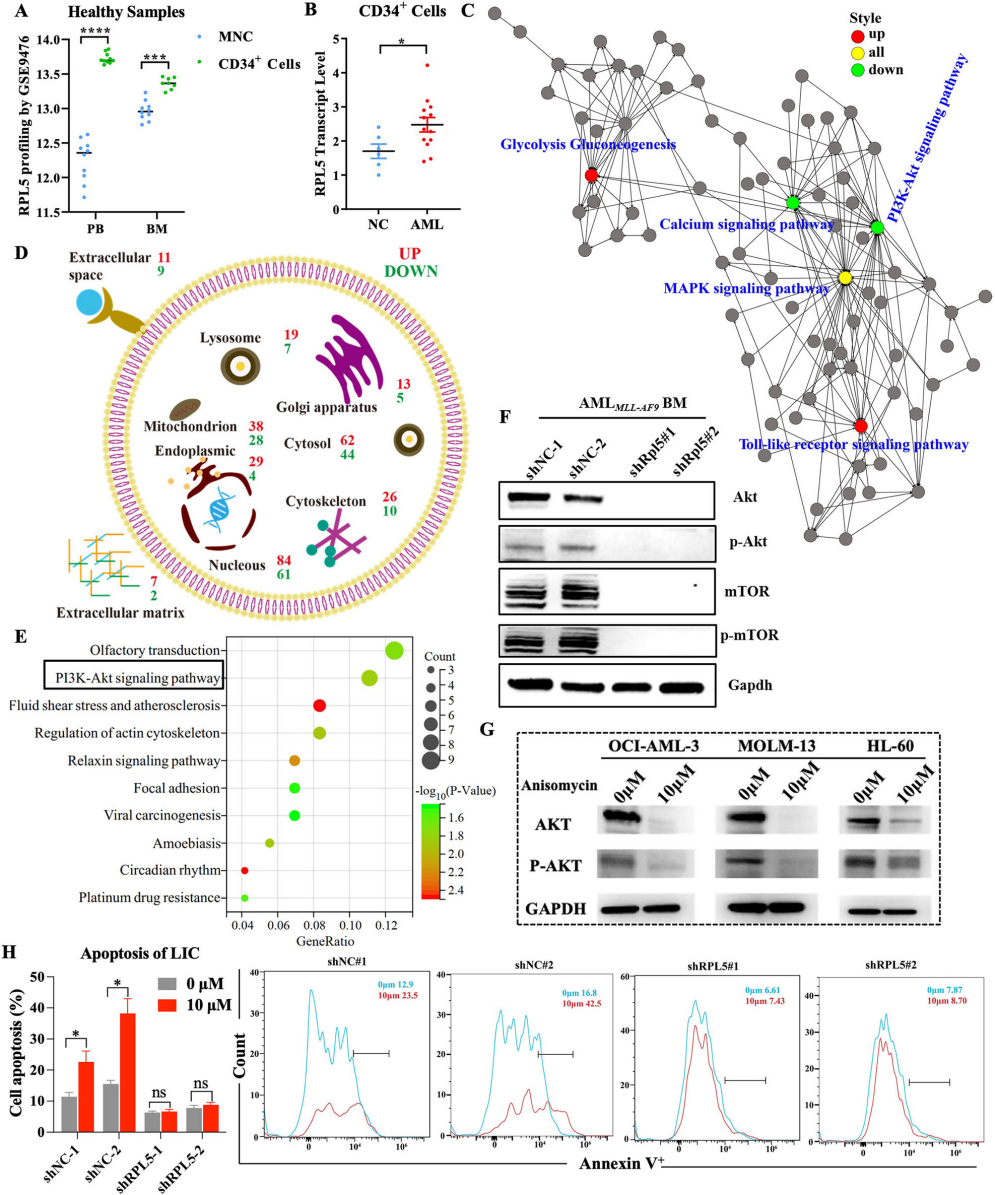
*RPL5* deficiency activates ribosomal stress-surveillance. **A** Levels of RPL5 mRNA in mature cells and CD34^+^ cells from PB (*n* = 10 vs. *n* = 10) or BM (*n* = 10 vs. *n* = 8) of healthy volunteers. **B** Levels of RPL5 mRNA in CD34^+^ cells from AML patients (*n* = 13) and age-matched normal controls (*n* = 6) of WMU-Cohort. **C** Path-act-network analysis of RNA-seq data. **D** Subcellular location of differentially expressed gene in proteomics. **E** KEGG pathway analysis of RPL5 based on the TCGA-LAML database. **F** Protein levels of Akt, p-Akt, mTOR, and p-mTOR in the BM of AML mice expressing shNC or shRpl5. **G** Protein levels of Akt and p-Akt in AML cells treated with Anisomycin. **H** The analysis of apoptotic LIC treated with the indicated dose of rapamycin (*n* = 3/group). (*P < 0.05, ***P < 0.001, ****P < 0.0001).

To investigate the selectivity of RPL5 deficiency on stem cells, we analyzed RPL5 expression specifically in CD34^+^ marked stem cells. The database of GSE9476 exhibited that RPL5 was higher expressed in CD34^+^ stem cells than MNC in PB and BM of healthy volunteers (Fig. 6A). Apart from RPL5, other ribosomal proteins, such as RPL3, RPL12, RPL29, RPS6, and RPS9, exhibited remarkably differential expression, which could be exploited in future research (Fig. S6A). Whereas the mRNA levels of RPL5 were highest in CD34^+^ marked LSCs compared with CD34^+^ marked healthy controls in the WMU-cohort (purity >90%), explaining the targeted effect of RPL5 on LSCs to some extent (Figs. 6B and S6B).

Additionally, we performed RNA-seq and label-free proteomics using BM cells from AML mice expressing shRpl5 or shNC to further understand the mechanism underlying Rpl5-regulated LSCs. The pathway-act-network analysis of RNA-seq data revealed a generalized role of Rpl5 in regulating 81 signaling pathways, including down-regulated PI3K-Akt pathway and disturbed mitogen-activated protein kinases (MAPK) signaling pathway (Fig. 6C). The MAPK pathway is at the nexus of numerous stress responses, among these the ribotoxic stress response (RSR) [34]. The gene set enrichment analysis also verified inhibited ribosome and ribosome biogenesis in eukaryotes in the shRpl5 group compared with the shNC group (Fig. S6C). In addition, the subcellular locations of differential proteins detected in the proteomics upon Rpl5 depletion were involved with almost all organelles, such as mitochondrion, lysosome, Golgi apparatus, and endoplasmic reticulum (Fig. 6D). All this reconfirmed the viewpoint that Rpl5 depletion eradicated LSC due to a cellular stress response rather than a specific function relating to Rpl5.

Apart from the RNA-seq data shown above (Fig. 6C), the KEGG pathway analysis based on the Pearson correlation algorithm (∣COR∣ > 0.4, *P* < 0.05) of the TCGA-LAML database also emphasized the role of RPL5 in regulating the PI3K-Akt signaling pathway (Fig. 6E). Therefore, we determined the protein levels of Akt and phosphorylated Akt (p-Akt) and found that they were much lower in the shRpl5 group than in the shNC group (Fig. 6F). The high-dose Anisomycin, which inhibited RPL5 in MOLM-13 cells, also inhibited the Akt and p-Akt protein levels (Fig. 6G). mTOR is a conserved downstream of the PI3K-Akt pathway, which connects ribosome biosynthesis with the cellular environment, thereby regulating nutrient uptake, hormone production, and cellular stress [35]. The total mTOR and phosphorylated mTOR (p-mTOR) were also decreased in the shRpl5 group compared with the shNC group (Fig. 6F). Consistently, rapamycin, a specific inhibitor of mTOR, induced cell apoptosis in LIC expressing shNC at 10 μM. However, they were unable to induce more apoptosis in LIC cells expressing shRpl5 (Fig. 6H). Therefore, RPL5 deficiency inhibited the PI3K-AKT-mTOR signaling pathway. Whereas Ras, one of the upstream PI3K-Akt signaling pathways that regulated MAPK [36, 37], was unchanged in the shRpl5 group compared with the shNC group (Fig. S6D).

### RPL5 deficiency-induced ribosomal stress alters branch-chain amino acid metabolism

**Fig. 7:**
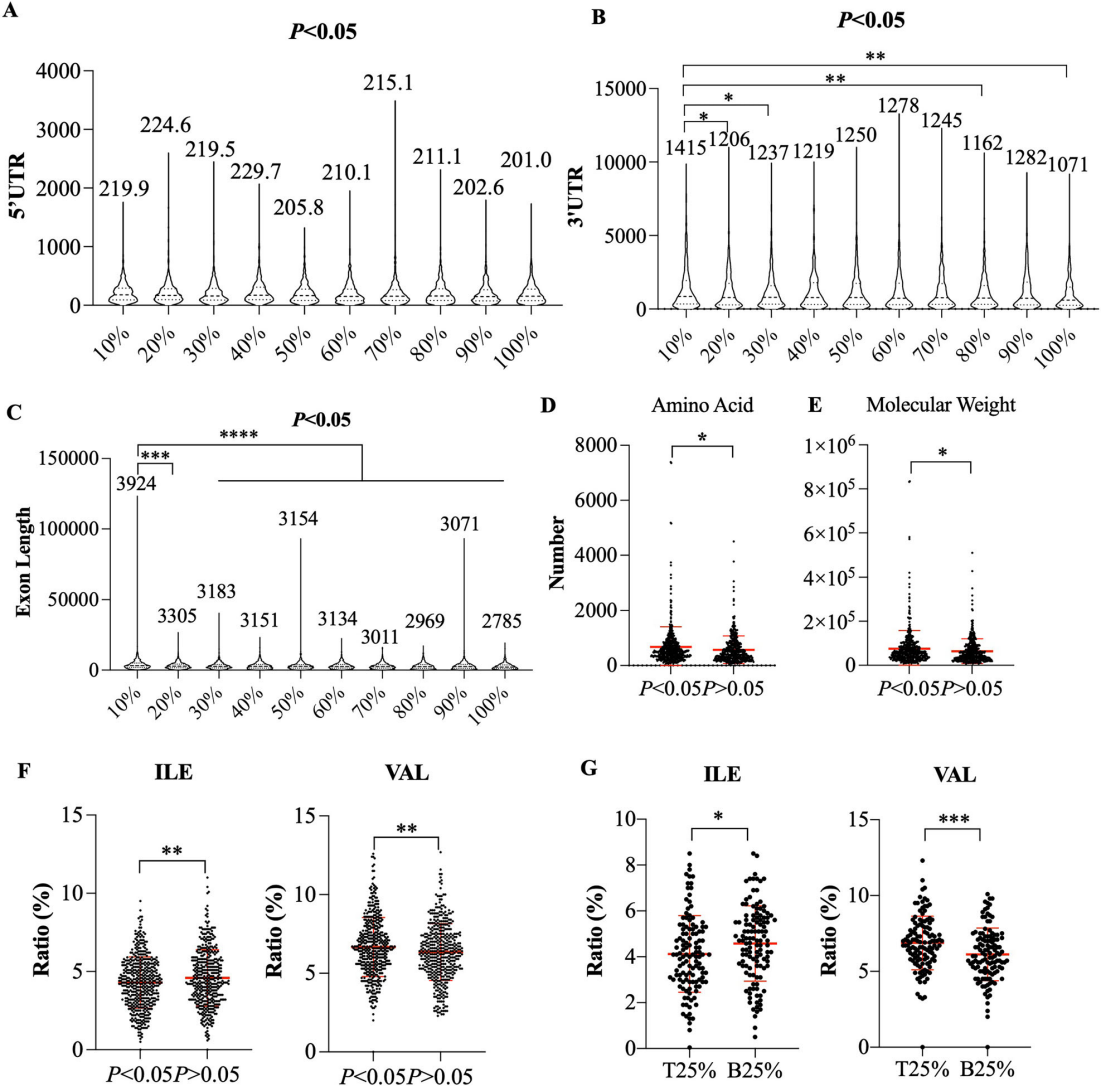
Proteins with a lower ratio of Ile and a higher ratio of Val are more sensitive to Rpl5 deficiency-induced ribosomal stress. **A** The variation of 5’UTR length in ten aliquots ranked from the lowest *P*-value to 0.05 (*n* = 904 to 905/group). **B** The variation of 3’UTR length in ten aliquots ranked from the lowest *P*-value to 0.05 (*n* = 904 to 905/group). **C** The variation of exon length in ten aliquots ranked from the lowest *P*-value to 0.05 (*n* = 904 to 905/group). The comparison of amino acid number (**D**) and molecular weight (**E**) of proteins with *P* < 0.05 (*n* = 478) or proteins with *P* > 0.05 (*n* = 446) in proteomics. **F** The comparison of the amino acid ratio of proteins with *P* < 0.05 (*n* = 478) or proteins with *P* > 0.05 (*n* = 446) in proteomics. **G** The comparison of amino acid ratios of proteins with *P* < 0.05, top 25% of highest log2|fold change| (T25%) and proteins with *P* < 0.05, bottom 25% of lowest log2|fold change| (B25%) (*n* = 120/group). (*P < 0.05, **P < 0.01, ***P < 0.001).

Ribosomal stress, a type of cellular stress, and its specific impact on the PI3K-Akt-mTOR signaling pathway sparked our curiosity. It has been shown that when ribosome levels, including RPL5, were reduced in patients with DBA, there was a more significant impact on transcripts with short or unstructured 5’UTRs, resulting in selectivity [38]. To examine whether transcripts with short or unstructured 5’UTRs were more sensitive to RPL5 deficiency-induced ribosomal stress, we analyzed the structure of differentially expressed transcripts in the RNA-seq (*P* < 0.05). All transcripts were ranked and evenly divided into ten aliquots based on *P*-values, with the 10% group containing the data with the smallest *P*-value and the 100% group containing the data with the closest *P*-value of 0.05. The length of 5’UTR and 3’UTR was varied and disordered among ten groups (Figs. 7A, B and S7A), while the exon length was much longer in the group with the smallest *P*-values compared with other groups (Fig. 7C). This tendency did not appear in non-differentially expressed genes (Fig. S7B). Similarly, the top 2.5% of transcripts have longer exon lengths compared to the bottom 2.5% of transcripts in the group with *P* < 0.05 but not in the group with *P* > 0.05 (Fig. S7C).

Correspondingly, the amino acid number and molecular weight of proteins with *P* < 0.05 in the proteomics were bigger than those of proteins with *P* > 0.05 (Fig. 7D, E). Furthermore, we analyzed the amino acid composition of proteins and found that the proteins with smaller *P*-values, which were more sensitive to ribosomal stress, had a lower isoleucine (Ile) to valine (Val) ratio when compared with the proteins with larger *P*-values (Figs. 7F and S8A). Ile and Val are the only two glycogenic BCAAs [39]; their specific changes also explain the effect of RPL5 deficiency on glucose metabolism (Fig. 6C). BCAAs regulate many signaling pathways, the most classic of which is the activation of the mTOR signaling pathway [40]. When we ranked proteins based on fold change in the *P* < 0.05 group, the Ile ratio of proteins with larger fold changes (Top 25%) was smaller than that of proteins with minor fold changes (Bottom 25%). Meanwhile, the Val ratio of proteins with larger fold changes (Top 25%) was bigger than that of proteins with minor fold changes (Bottom 25%) (Fig. 7G). Therefore, RPL5 depletion-induced ribosomal stress disrupted stemness maintenance by affecting BCAA metabolism, specifically inhibiting the PI3K-AKT-mTOR signaling pathway.

## Discussion

Ribosomal proteins not only determine protein biosynthesis but also participate in DNA repair, cell development regulation, and other extra-ribosomal functions [7, 8]. RPL5, a constitutive element of the 5S ribonucleoprotein complex, was highly expressed in CD34^+^ marked stem cells, particularly in LSCs. Silencing RPL5 with RNA interference inhibited AML cell survival in vitro and in vivo, and significantly impaired the colony-forming ability of LSCs, demonstrating selectivity toward LSCs. The specific eradication of LSCs was considered a therapeutic strategy for relapsed AML and drug resistance. Therefore, RPL5 deficiency impeded AML progression and extended the survival of AML mice in the long term.

Previous studies have reported that RPL5 regulates cell fate primarily by stabilizing p53 or degrading c-Myc in solid tumors [30, 31]. However, when HEATR3 destabilized RPL5/RPL11 and induced DBA, it did not activate the p53 signaling pathway [41]. The presence of p53 mutations and the absence of MDM2/HDM2 in some tumor cells also confirmed the existence of a p53-independent pathway in RPL5 mutation-induced cell apoptosis [18]. In this study, the RNA-seq and proteomic data did not reveal clues relating to p53 or c-Myc but instead highlighted the PI3K-Akt signaling pathway. The PI3K-Akt signaling pathway participated in the maintenance of HSPCs and was highly activated in many cancers, including AML [36, 42]. Consistent activation of Akt in HSPCs leads to hematopoietic transformation. A few studies have mentioned that the activation of the classical p53 pathway by spindle1 or HEATR1, via RPL5, also involves regulation of the PI3K-Akt pathway [30, 43]. However, they have not systematically explored RPL5-related molecular mechanisms, particularly in AML.

Moreover, we found that the selective eradication of LSC induced by RPL5 deficiency was not due to a disruption of the specific function related to RPL5, but instead to a cellular stress response. Ribosomal stress, also known as nucleolar stress, occurs in cells with disturbed ribosome biosynthesis [41]. The activation of ribosomal stress surveillance, such as RSR, ribosome-associated quality control, and the integrated stress response (ISR), determines cell fate [34]. Previous studies have reported that modest ribosomal stress stimulates an ISR, enabling cells to survive, while severe ribosomal stress triggers an RSR, leading to cell apoptosis [32, 33]. Considering the MAPK pathway is at the nexus of RSR [34] and only high-dose Anisomycin affected RPL5 expression, RPL5 deficiency in this study was more likely to induce severe ribosomal stress in LSCs. Additionally, the PI3K-Akt and MAPK pathways were synchronously activated in HSPCs during emergency hematopoiesis, which may explain the unaffected colony formation observed upon Rpl5 deficiency in HSPCs [44]. MAPK signaling pathways also regulated the response of LSC to therapies [45, 46].

Unlike previous reports that transcripts with short or unstructured 5’UTRs were more sensitive to reduced ribosome levels in patients with DBA [38], we found that transcripts with longer exon lengths were more sensitive to RPL5 deficiency-induced ribosomal stress, generating selectivity. Specifically, proteins with a lower Ile to Val ratio exhibited greater changes upon RPL5 deficiency-induced ribosomal stress. Ile and Val are two glycogenic BCAAs, and BCAA metabolism affects glycometabolism, as well as innate and adaptive immune responses. All these characters were observed in the multi-omics sequencing (data not shown). Besides, altered PI3K-Akt protein expression was observed in hydrophobic BCAA peptides, which induced a hypoglycemic effect [47, 48]. BCAA also regulated the activation of the mTOR signaling pathway, and the translational co-regulation of ribosomal proteins was demonstrated downstream of mTOR signaling [49]. Therefore, mTOR, as a conserved downstream of the PI3K-Akt pathway, connects ribosome biosynthesis with the cellular environment, thereby regulating nutrient uptake, hormone production, and cellular stress [35].

## Conclusions

RPL5 was highly expressed in leukemia cells and was required for AML cell survival. It was most highly expressed in LSCs, and RPL5 deficiency selectively eradicated LSCs. The cellular mechanism revealed that RPL5 deficiency impaired LSC frequency and function through a G0-phase cell cycle arrest. Meanwhile, the study of molecular mechanisms found that *RPL5* deficiency-induced ribosomal stress specifically affected transcripts with longer exon length and proteins with a lower Ile to Val ratio. Ile and Val, as glycogenic BCAAs, their altered metabolism disrupted the PI3K-Akt-mTOR signaling pathway, which has been highlighted in the multi-omics sequencing. Moreover, the activated RSR ribosomal stress-surveillance induced by severe ribosomal stress also contributed to the elimination of LSC. In short, RPL5 could be developed as a therapeutic target to benefit AML patients with relapse and drug resistance.

## Supplementary information

Supplementary Figures and Tables

WB original figures

## Data Availability

All data generated or analyzed from this study are available within this article and its Supplementary materials. Sequencing data and R scripts supporting the results reported in this paper are archived in the GitHub (https://github.com) public repository, Fang-He1111/RPL5. Further information and requests for resources and reagents should be directed to and will be fulfilled by the lead contact, Shenmeng Gao (gaoshenmeng77@wzhospital.cn).
