# Supplementary material for: RPL5 deficiency-induced ribosomal stress targets a select subset of proteins and inhibits the PI3K-Akt-mTOR signaling pathway to eradicate leukemia stem cells
Source: Cell Death Dis. 2025 Dec 18;17(1):117. doi: 10.1038/s41419-025-08379-1 (PMC12847885; doi:10.1038/s41419-025-08379-1)
Supplement: Supplementary file 1 — Supplementary Figures and Tables [file 41419_2025_8379_MOESM1_ESM.pdf]

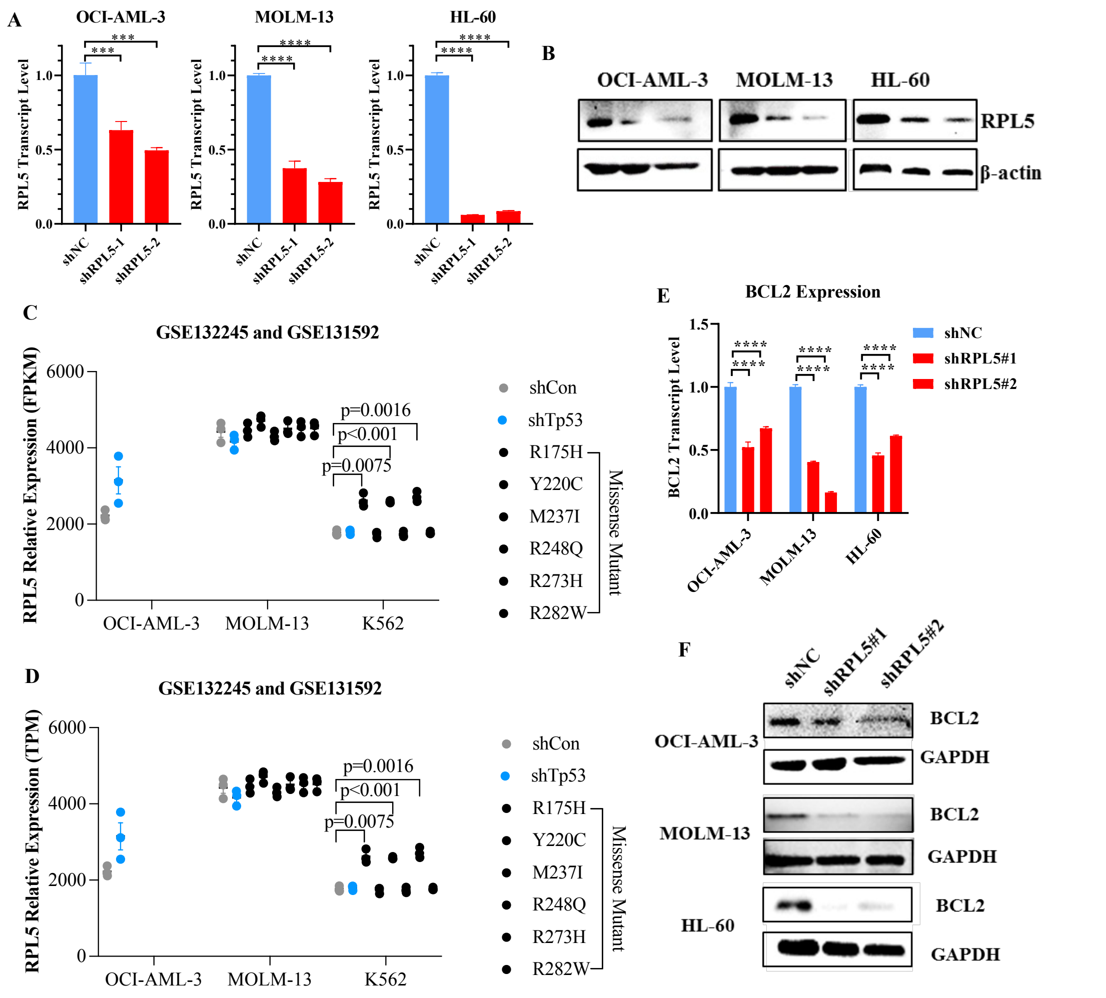

Fig. S1 (A-B) Levels of *RPL5* mRNA (A) and protein (B) in AML cell lines expressing shNC or shRPL5 (n = 3). (C-D) Levels of RPL5 expression in AML cell lines expressing shNC, shp53 or missense mutation. (E-F) The mRNA (E) and protein levels (F) of BCL2 in AML cell lines expressing shNC or shRPL5 (n = 3). (*** $P < 0.001$, **** $P < 0.0001$)

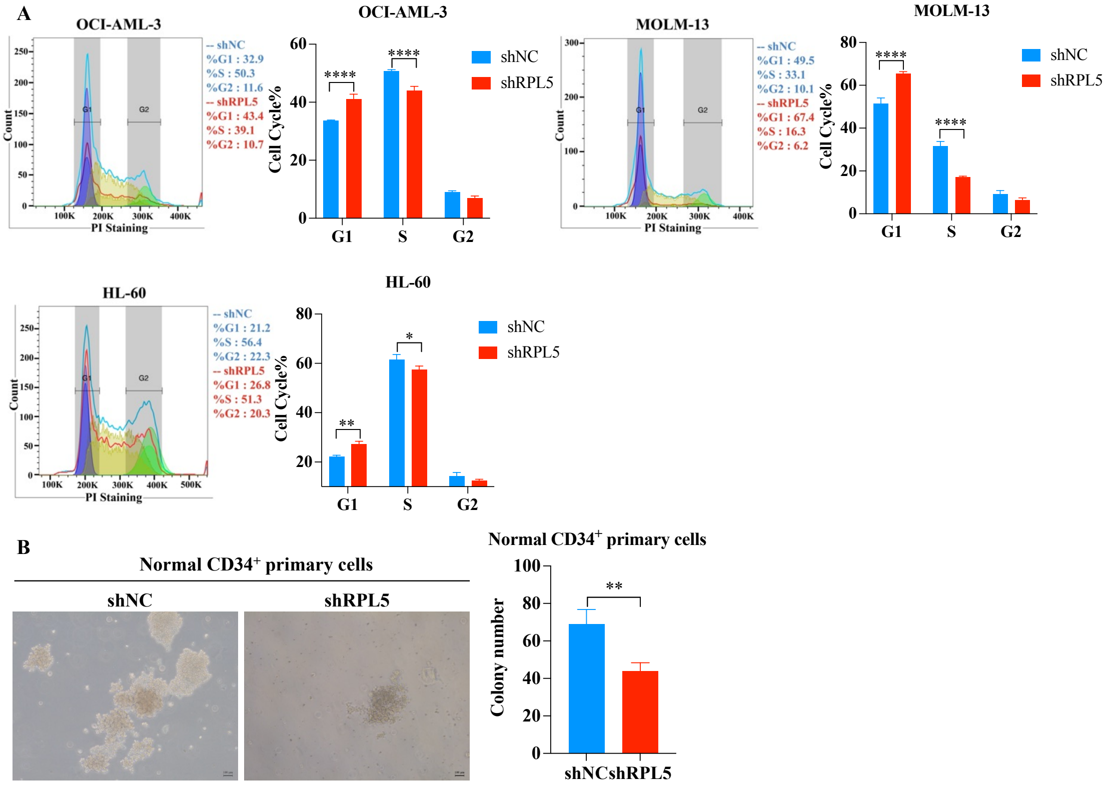

Fig. S2 (A) Cell cycle of AML cell lines expressing shNC or shRPL5 (n = 3). (B) Colony formation of normal CD34$^+$ primary cells expressing shNC or shRPL5 (n = 3). (*$P < 0.05$, ** $P < 0.01$, *** $P < 0.001$, **** $P < 0.0001$)

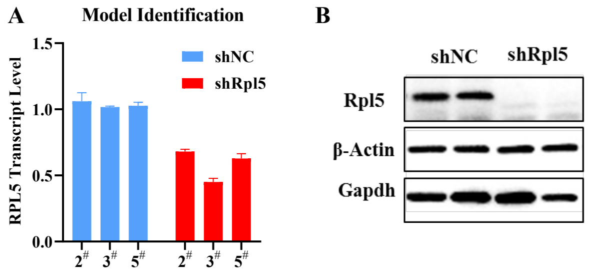

Fig. S3 (A-B) Levels of Rpl5 mRNA (A) and protein (B) in AML murine model expressing shNC or shRpl5 (n = 3).

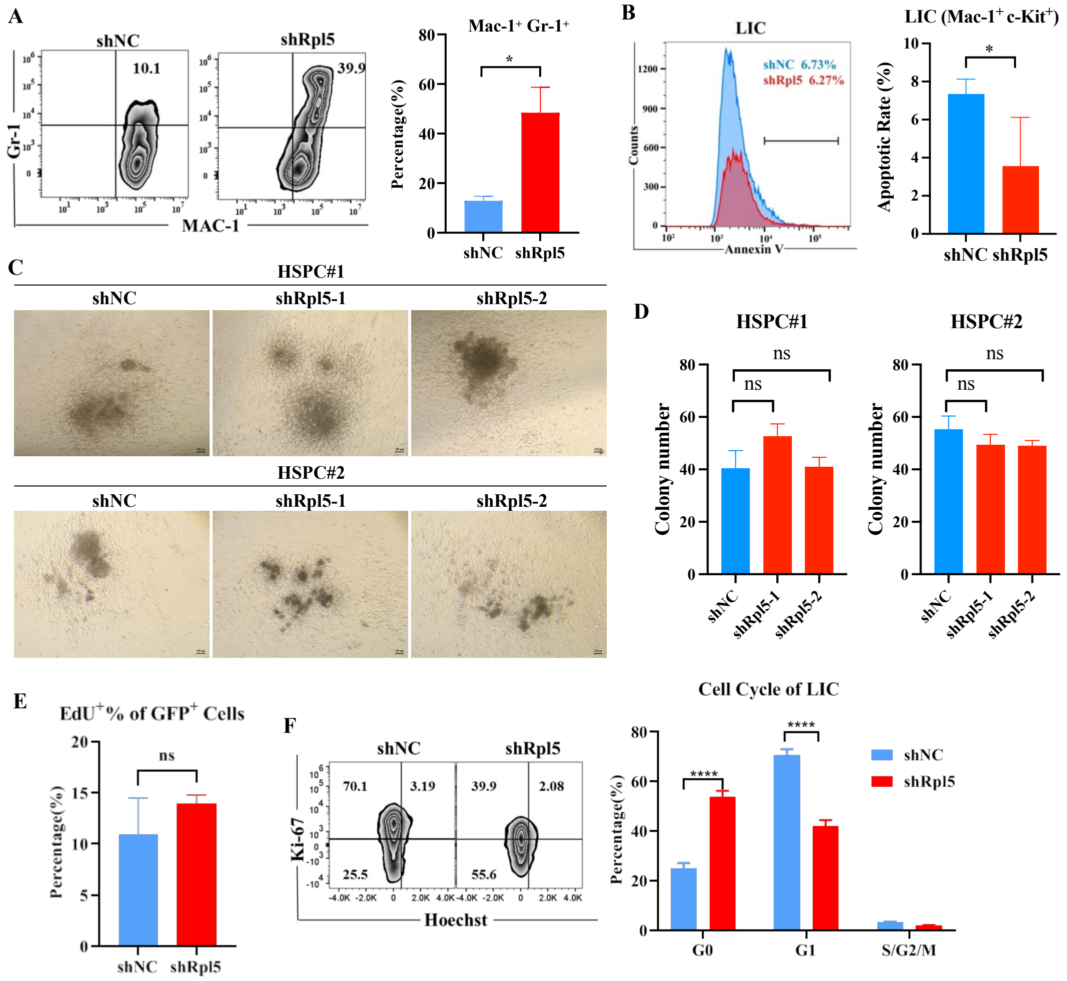

Fig. S4 (A) Representative picture and the flow cytometric analysis of differentiated AML cells in BM from secondary recipients expressing shNC or shRpl5 (n = 4). (B) The representative picture and the flow cytometric analysis of the cell apoptosis of BM LICs expressing shNC or shRpl5 (n = 6). (C, D) Representative figure (C) and colony number (D) produced by lineage-negative cells from normal HSPCs. (E) The percentage of EdU$^+$ BM GFP$^+$ cells from secondary recipients expressing shNC or shRpl5 (n = 4). (F) The representative picture and the flow cytometric analysis of the cell cycle of BM LICs from secondary recipients expressing shNC or shRpl5 (n = 4). (*$P < 0.05$, **** $P < 0.0001$, ns = no significance)

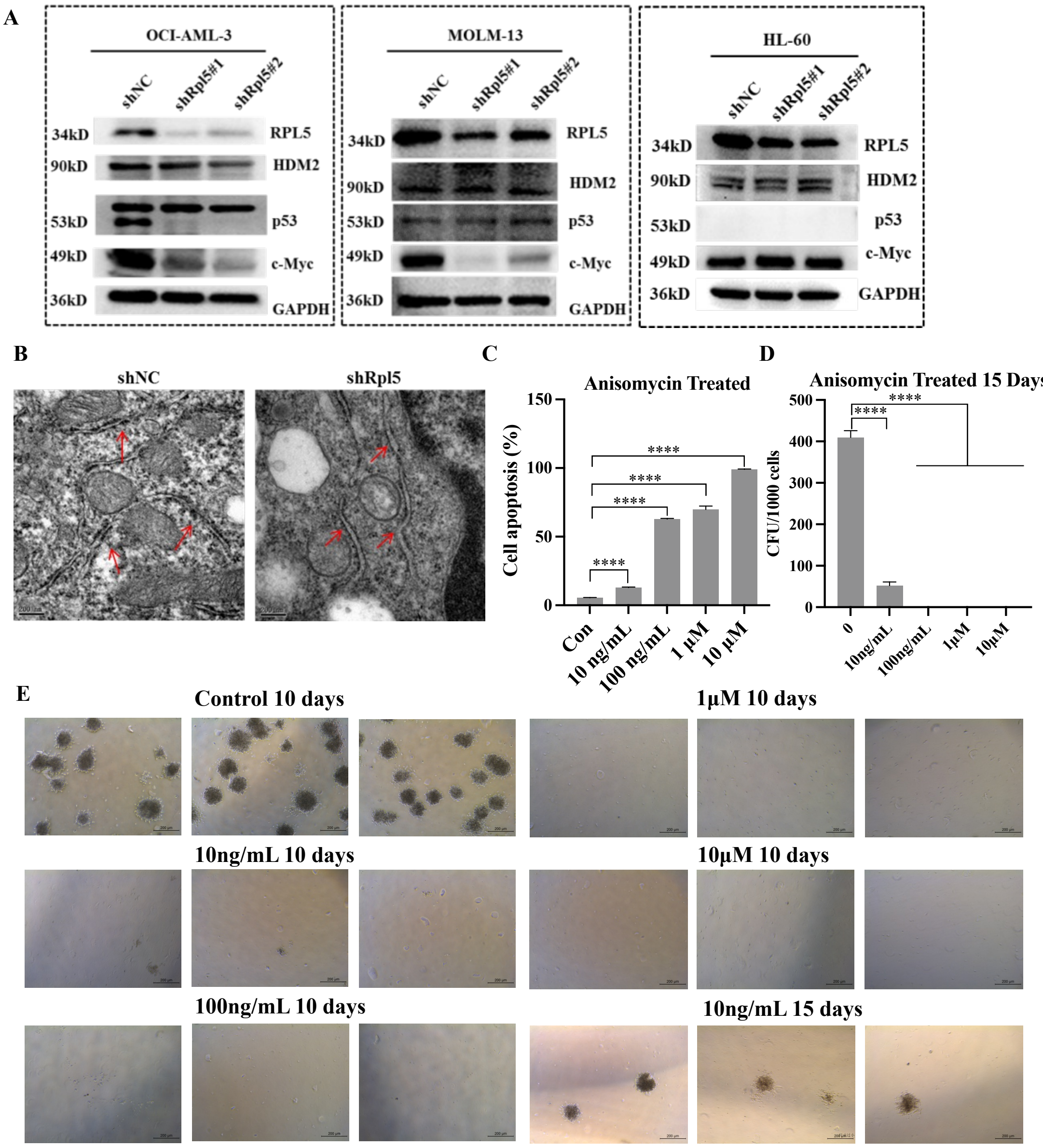

Fig. S5 (A) Levels of HDM2, p53 and c-Myc proteins in AML cells expressing shNC or shRPL5. (B) TEM picture of GFP^+ BM cells from secondary recipients expressing shNC or shRpl5. (C) Flow cytometric analysis of cell apoptosis induced by Anisomycin treatment in MOLM-13 cells (n = 4). (C-D) Colonies forming (C) and the representative picture (D) of MOLM-13 cells treated with Anisomycin (n = 3). (**** $P < 0.0001$)

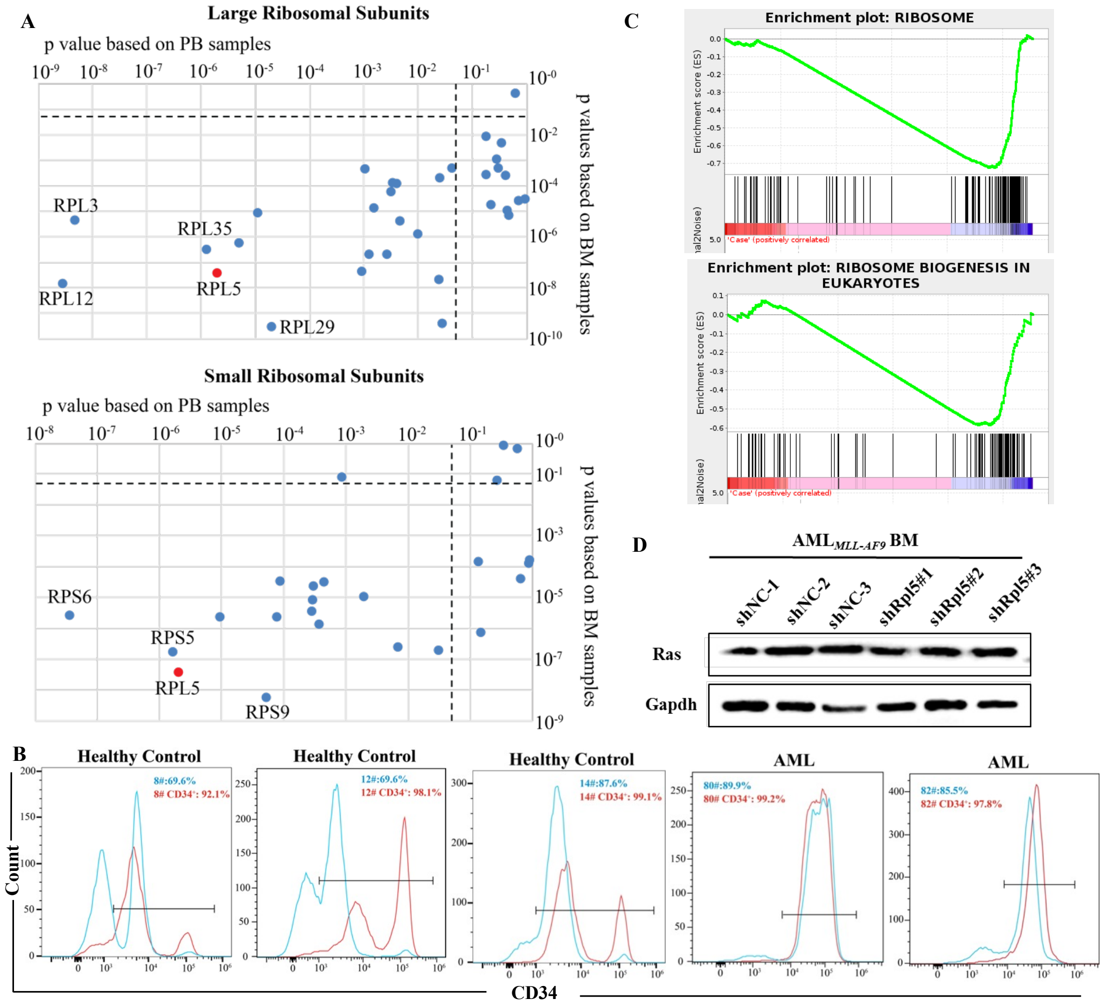

Fig. S6 (A) *P*-values of CD34$^+$ cells vs. MNC in PB and BM samples based on GSE9476. (B) Representative picture of CD34$^+$ cell sorting with primary cells. (C) GSEA relating to ribosomal stress based on RNA-seq data of secondary recipients expressing shNC or shRpl5. (D) Protein levels of Ras in BM of AML mice expressing shNC or shRpl5.

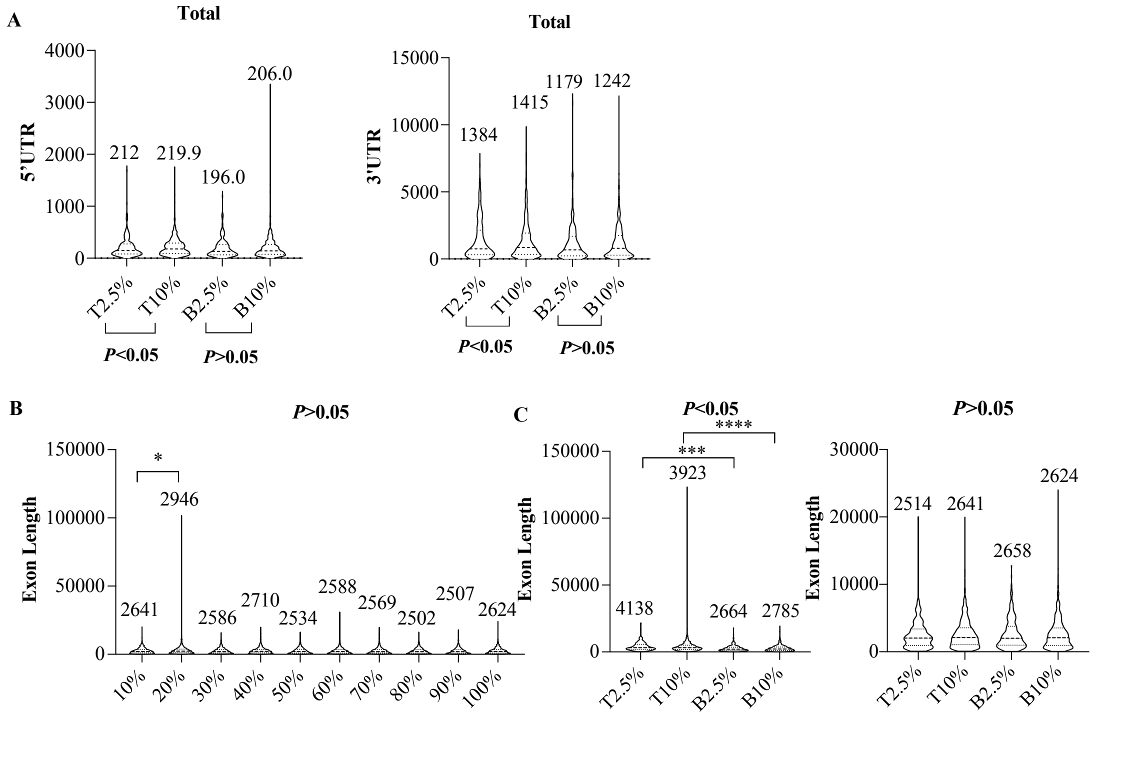

Fig. S7 (A) The comparison of 5'UTR and 3'UTR of transcripts with the lowest *P*-values (T2.5% n = 225, T10% n = 903) or the highest *P*-values (B2.5% n = 225, B10% n = 903). (B) The variation of exon lengths in ten aliquots ranked from 0.05 to the highest *P*-value (n = 1066 to 1067). (C) The comparison of exon lengths of transcripts with the lowest *P*-values and highest *P*-values in $P < 0.05$ group (T2.5% or B2.5% n = 225, T10% or B10% n = 903) or $P > 0.05$ group, respectively. (T2.5% or B2.5% n = 266, T10% or B10% n = 1067). (*$P < 0.05$, ***$P < 0.001$, ****$P < 0.0001$).

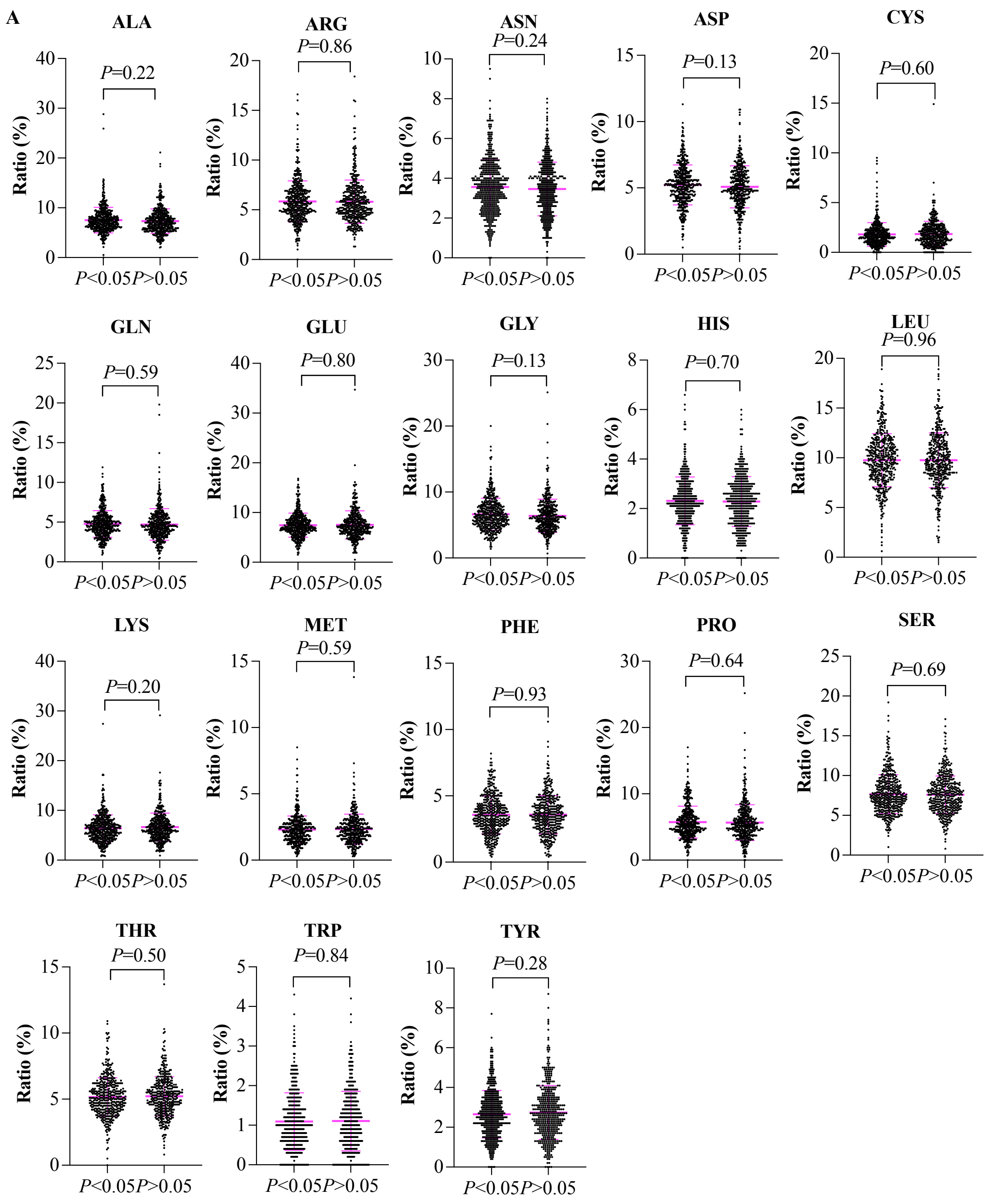

Fig. S8 (A) The comparison of amino acid ratio of proteins with $P < 0.05$ (n = 478) or $P > 0.05$ (n = 446) in proteomics.

Table S1. The clinical characteristics of 74 AML patients

Characteristic	All patients N (%)
Overall	74
**Gender**	
Male	40(54.1)
Female	34(45.9)
Age (range)	54(21-73)
**FAB subtype**	
M1	7(9.5)
M2	12(16.2)
M3	6(8.1)
M4	20(27.0)
M5	28(37.8)
Unclassified	1(1.4)
**Cytogenetics**	
Normal karyotype	32(43.2)
t (15;17)	6(8.1)
t (8;21)	12(16.2)
t (11q23)	6(8.1)
Inv (16)	7(9.5)
+8	4(5.4)
+11	2(2.7)
+21	1(1.4)
Complex karyotype	2(2.7)
NA	2(2.7)
**Molecular genetic abnormality**	
PML-RARα	6(8.1)
AML1-ETO	12(16.2)
CBF-MYH11	7(9.5)
MLL-arrangements	6(8.1)
WT1 mutation	8(10.8)
FLT3 mutation	9(12.2)
CEBPα mutation	11(14.9)
TET2 mutation	7(9.5)
MLL-PTD mutation	2(2.7)
c-Kit mutation	6(8.1)
NPM1 mutation	4(5.4)

Table S2. Antibodies

Name	Catalog	Brand
RPL5	14568S	CST
GAPDH	Ab6276	Abcam
BCL2	Ab182858	Abcam
Phospho-Akt1(Ser473)	ET1607-73	HUABIO
Akt1/2/3	ET1609-51	HUABIO
mTOR	2983S	CST
Phospho-mTOR (Ser2448)	5536S	CST
JNK1+JNK2+JNK3	ET1601-28	HUABIO
Phospho-JNK1/2/3(T183+T183+T221)	ET1609-42	HUABIO
p38	ET1702-65	HUABIO
Phospho-p38	ER2001-52	HUABIO
ERK1/2	ET1061-29	HUABIO
Eek1(pT202/pY204) + ERk2(pT185/pY187)	ET1610-13	HUABIO
ERK5	ET1612-7	HUABIO
Phospho-Erk5 (Thr218/Tyr220)	Q13164	CST
Meis1	ET1707-84	HUABIO
RAS	ET1702-94	HUABIO
β-actin	66009-1-lg	Proteintech

Table S3. Primers

**Genes**	**Sequences**
hGAPDH-F	5′- CCG GGA AAC TGT GGC GTG ATG G-3′
hGAPDH-R	5′-AGG TGG AGG AGT GGG TGT CGC TGT T-3′
hRPL5-F	5′-GCT CGG AAA CGC TTG GTG ATA-3′
hRPL5-R	5′-CCC TCT ATA CGG GCA TAA GCA AT-3′
hBCL2-F	5′-GGT GGG GTC ATG TGT GTG G-3′
hBCL2-R	5′-CGG TTC AGG TAC TCAG TCA TCC-3′
mRpl5-F	5′-GGC GGC GAG AGG GTA AAA C-3′
mRpl5-R	5′-GCA CAG ACG ATC ATA TCC CCT TC-3′
mCdkn2c-F	5′-GGG GAC CTA GAG CAA CTT ACT-3′
mCdkn2c-R	5′-AAA TTG GGA TTA GCA CCT CTG AG-3′
mCdk17-F	5′-AAC CTT GCA CGA CAT CGT TCA-3′
mCdk17-R	5′-CAC GGA GAA TTT GGT ACA GGA A-3′
mBcl2-F	5′-GTC GCT ACC GTC GTG ACT TC-3′
mBcl2-R	5′-CAG ACA TGC ACC TAC CCA GC-3′
murine β-actin-F	5′-GGC TGT ATT CCC CTC CAT CG-3′
murine β-actin-R	5′-CCA GTT GGT AAC AAT GCC ATG T-3′
*shRpl5 (murine)	5′-CGA ACT ACA ACT GGC AAT AAA-3′
*shRPL5#1 (human)	5′-GCC TAC TTT AAG AGA TAC CAA-3′
*shRPL5#2 (human)	5′-TTG GTA TCT CTT AAA GTA GGC-3′
*OE-RPL5-F (human)	5′ -GGA ATTC ATG GGG TTT GTT AAA GTT GT-3′
*OE-RPL5-R (human)	5′ -CGG GAT CCT TAG CTC TCA GCA GCC CGC TC-3′

*Primers for constructing plasmid

Table S4. TP53 mutation in AML cell lines

Cell Lines	Mutation
MOLM-13	NA
OCI-AML-3	NA
HL-60	TP53 deletion, Homozygous
U937	p.Val173Trpfs*59 (c.559+1G>A), Heterozygous
THP-1	p.Arg174fs*3 (c.520_545del26), Heterozygous
HEL	p.Met133Lys (c.398T>A), Homozygous
K562	p.Gln136fs*13 (c.406_407insC), Homozygous
Kasumi-1	p.Arg248Gln (c.743G>A), Homozygous

Table S5. Cell lines that RPL5 gene has at least one damaging mutation

A204	HUTU80	LO68	NZM11	SLR24	WM2644
ANGMCSS	JHOC5	MET2B	REH	SUPB 15	WSUFSCCL
DND41	LNCaP CLONE FGC	ML-2	SKMEL24	UACC257	

Table S6. ELDA analysis

**Quantification of LICs**	**Repopulation ratio**	
**Transplanted cells**	**shNC**	**shRpl5**
3	2/6	0/6
10	5/6	0/6
30	6/6	1/6
100	6/6	3/5
300	6/6	5/5
900	5/5	5/5
Frequencies of LICs	1:6.5	1:114
95% confidence interval	3.41-12.9	56.21-231.8
