# Supplementary material for: RPL5 deficiency-induced ribosomal stress targets a select subset of proteins and inhibits the PI3K-Akt-mTOR signaling pathway to eradicate leukemia stem cells
Source: Cell Death Dis. 2025 Dec 18;17(1):117. doi: 10.1038/s41419-025-08379-1 (PMC12847885; doi:10.1038/s41419-025-08379-1)
Supplement: Supplementary file 2 — WB original figures [file 41419_2025_8379_MOESM2_ESM.docx]

Fig.1(E) The representative WB picture of RPL5 protein levels in AML cell lines compared with BM MNC from healthy controls.


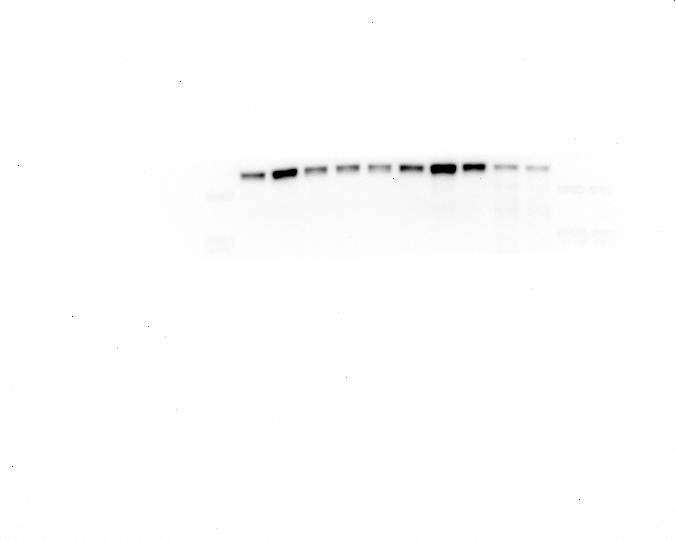


Rpl5


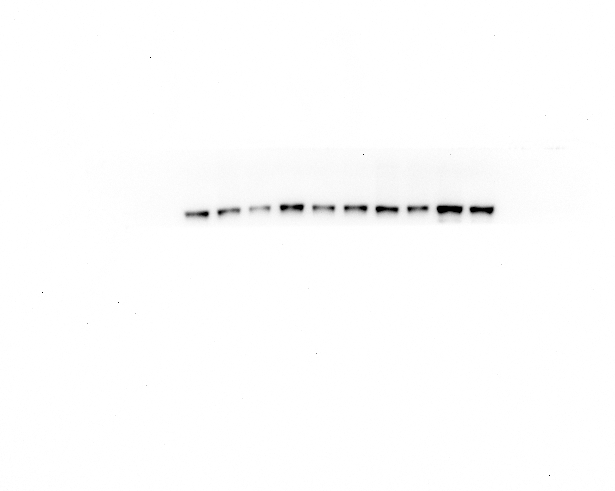


β-actin

Fig. 5 (E) RPL5 deficiency induces ribosomal stress.

OCI-AML-3 Puromycin GAPDH


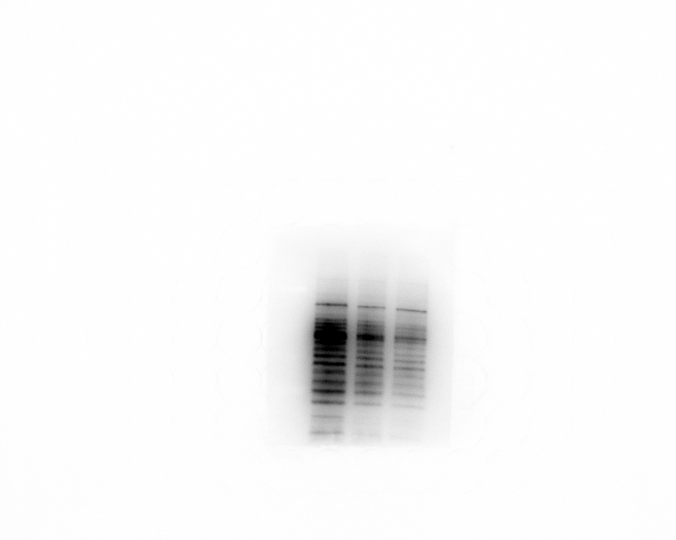

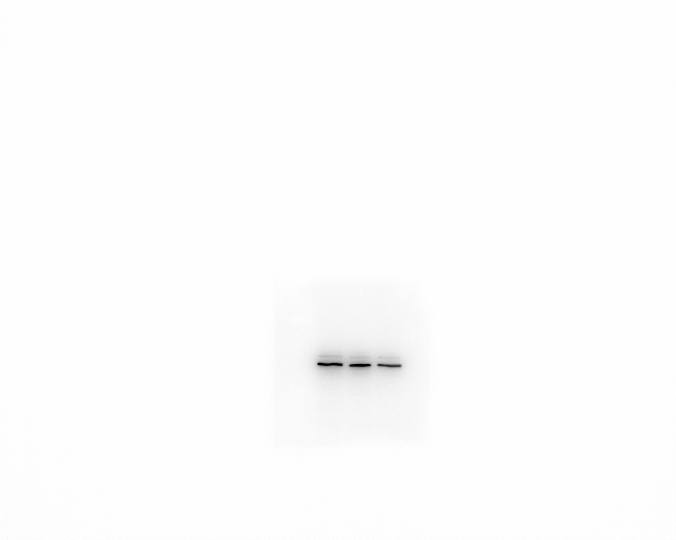


MOLM-13 Puromycin GAPDH


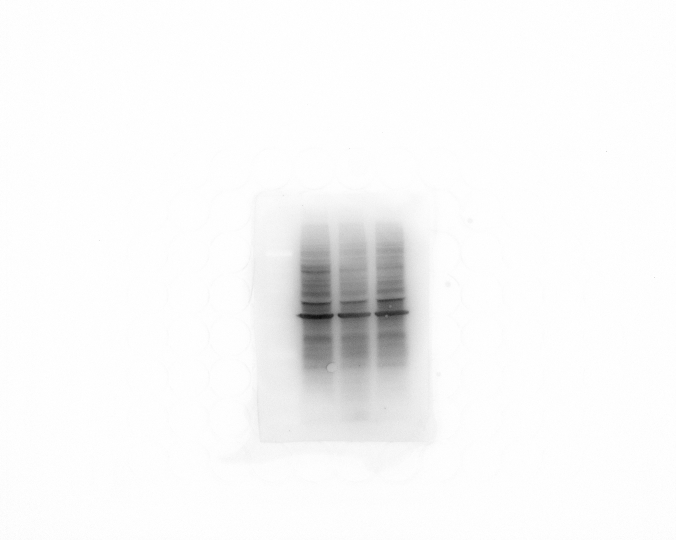

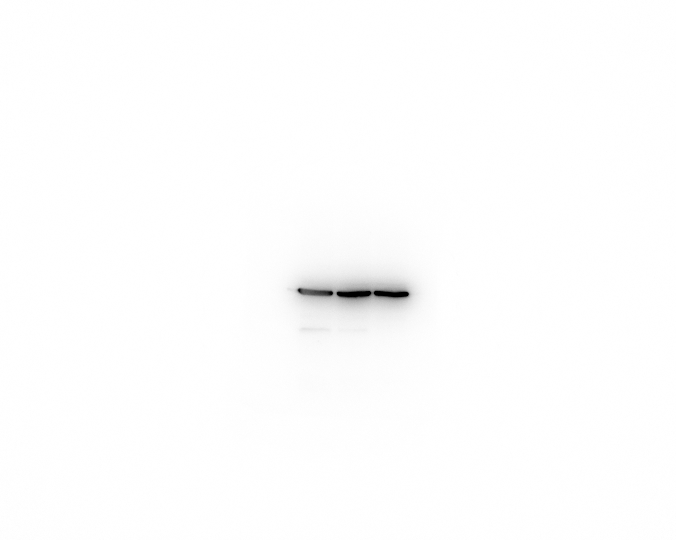


HL-60 Puromycin GAPDH


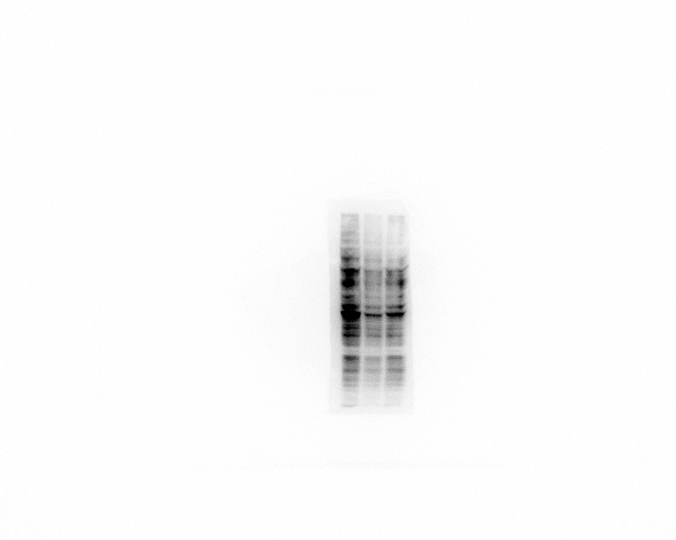

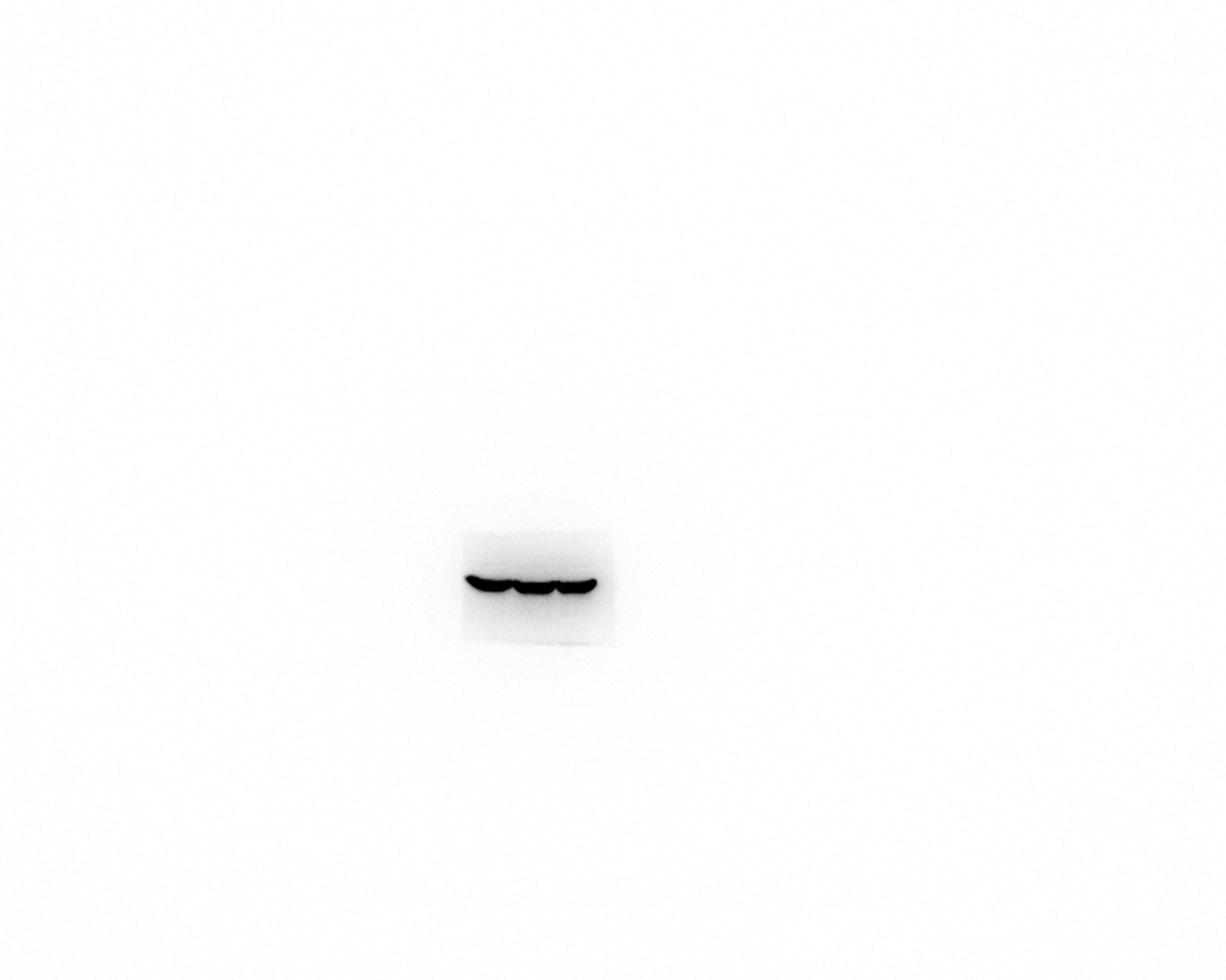


Figure 5 (G) The protein level of RPL5 in MOLM-13 cells treated with Anisomycin.


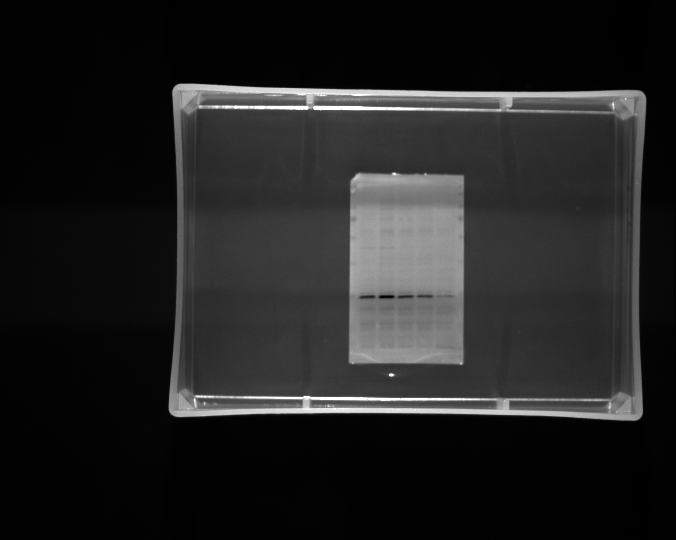

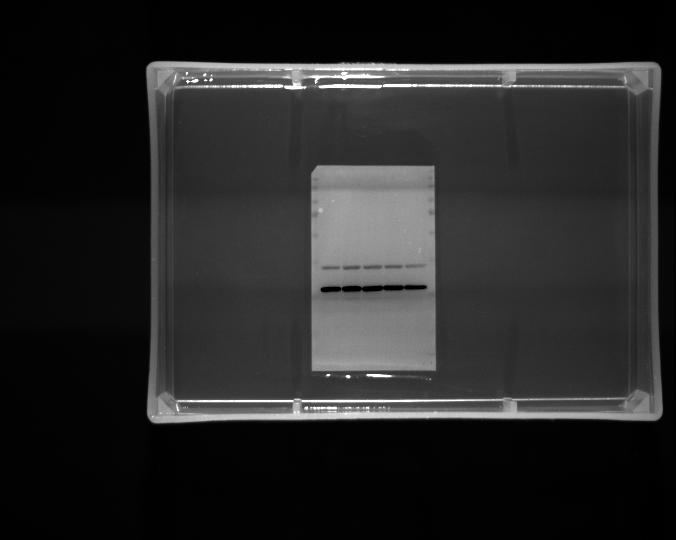


RPL5 β-actin and GAPDH

Fig. 6 (G) Protein levels of Akt, p-Akt in AML cell lines treated with Anisomycin.

OCI-AML-3 AKT P-AKT GAPDH


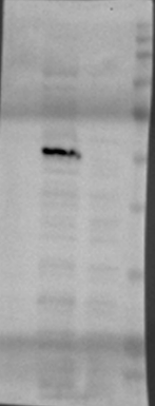

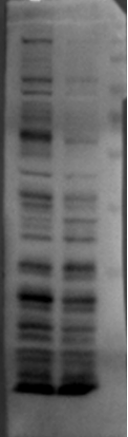

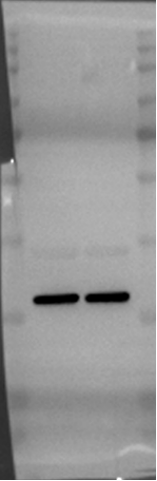


MOLM-13 AKT P-AKT GAPDH


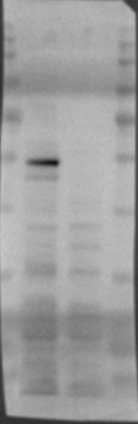

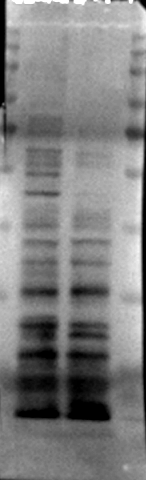

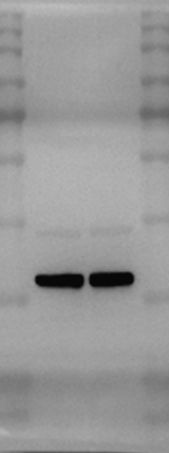


HL-60 AKT P-AKT GAPDH


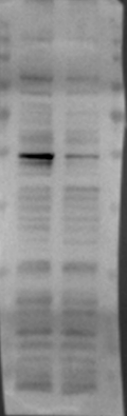

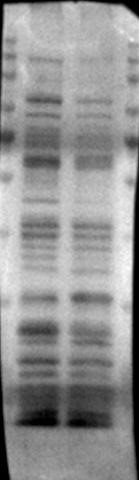

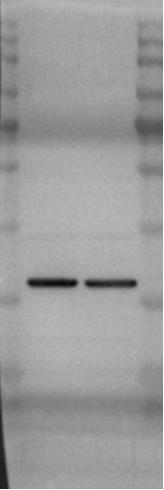


HL60

Molm-13

OCI-AML-3


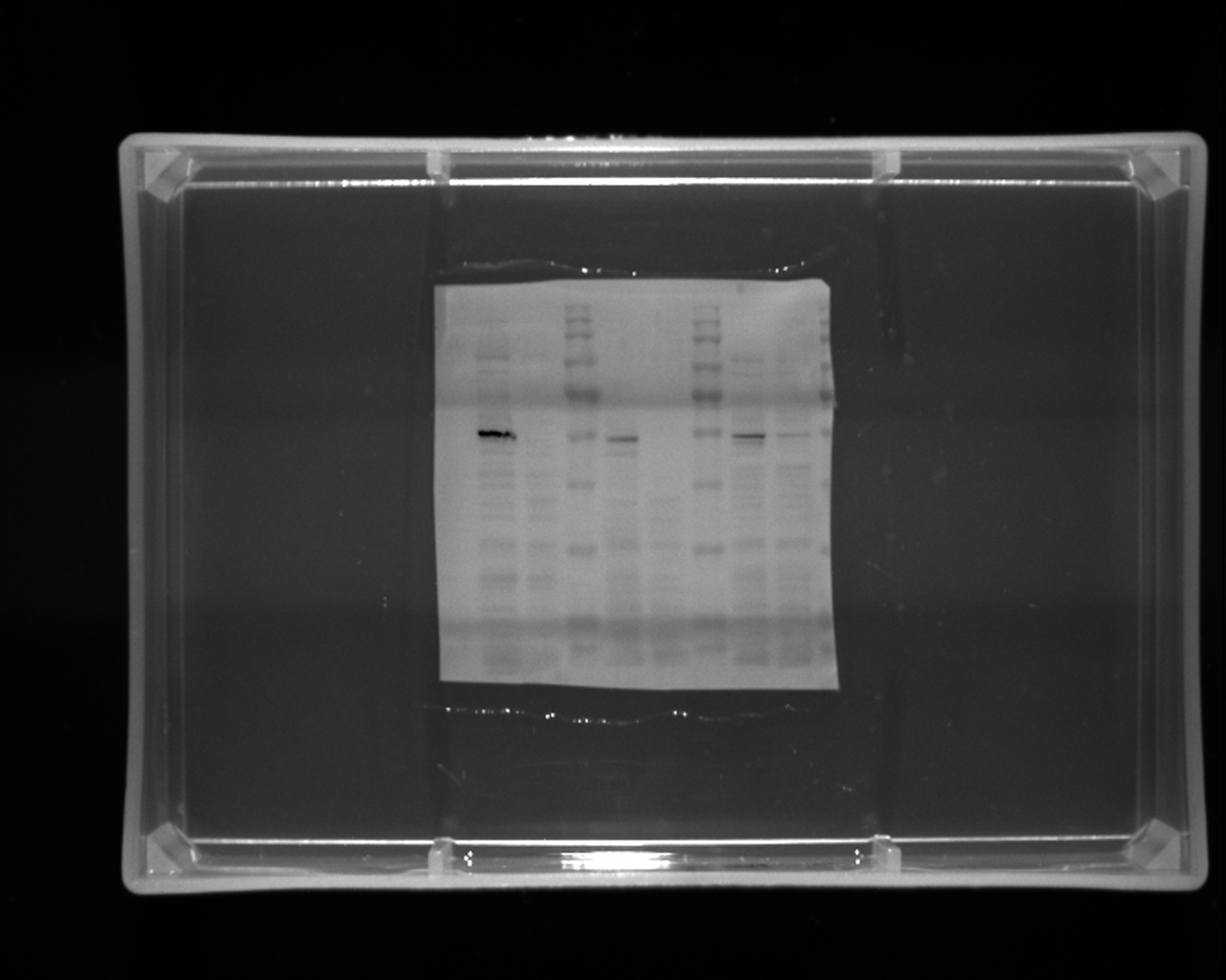


AKT


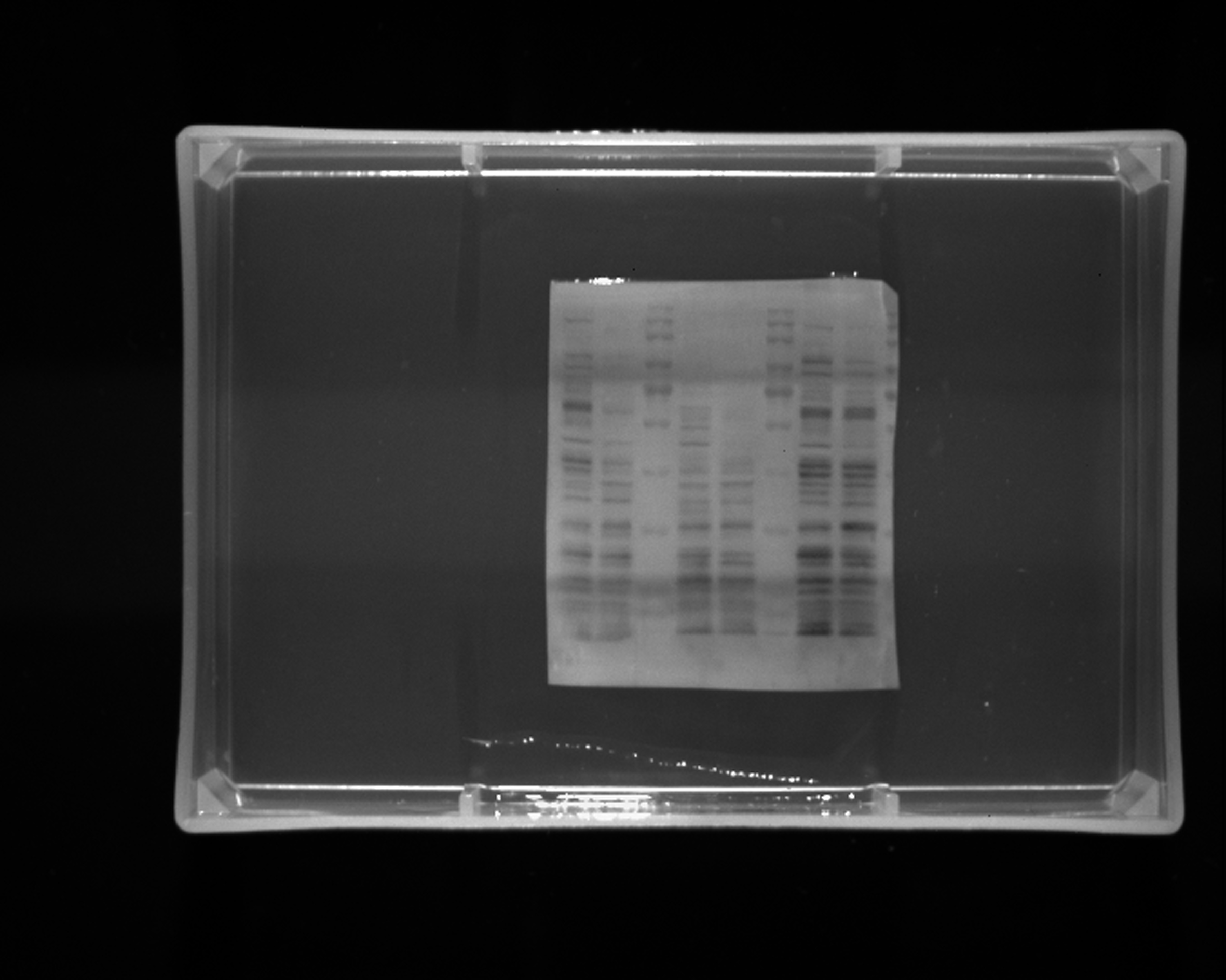


P-AKT
